# Detecting Mental Health Behaviors Using Mobile Interactions: Exploratory Study Focusing on Binge Eating

**DOI:** 10.2196/32146

**Published:** 2022-04-25

**Authors:** Julio Vega, Beth T Bell, Caitlin Taylor, Jue Xie, Heidi Ng, Mahsa Honary, Roisin McNaney

**Affiliations:** 1 Department of Medicine University of Pittsburgh Pittsburgh, PA United States; 2 University of York York United Kingdom; 3 York St John University York United Kingdom; 4 Department of Human Centred Computing Monash University Clayton Australia; 5 Lancaster University Lancaster United Kingdom

**Keywords:** eating disorder, binge eating, mental health, mobile sensing, context-aware computing, NAP, EMA, mobile phone

## Abstract

**Background:**

Binge eating is a subjective loss of control while eating, which leads to the consumption of large amounts of food. It can cause significant emotional distress and is often accompanied by purging behaviors (eg, meal skipping, overexercising, or vomiting).

**Objective:**

The aim of this study was to explore the potential of mobile sensing to detect indicators of binge-eating episodes, with a view toward informing the design of future context-aware mobile interventions.

**Methods:**

This study was conducted in 2 stages. The first involved the development of the DeMMI (Detecting Mental health behaviors using Mobile Interactions) app. As part of this, we conducted a consultation session to explore whether the types of sensor data we were proposing to capture were useful and appropriate, as well as to gather feedback on some specific app features relating to self-reporting. The second stage involved conducting a 6-week period of data collection with 10 participants experiencing binge eating (logging both their mood and episodes of binge eating) and 10 comparison participants (logging only mood). An optional interview was conducted after the study, which discussed their experience using the app, and 8 participants (n=3, 38% binge eating and n=5, 63% comparisons) consented.

**Results:**

The findings showed unique differences in the types of sensor data that were triangulated with the individuals’ episodes (with nearby Bluetooth devices, screen and app use features, mobility features, and mood scores showing relevance). Participants had a largely positive opinion about the app, its unobtrusive role, and its ease of use. Interacting with the app increased participants’ awareness of and reflection on their mood and phone usage patterns. Moreover, they expressed no privacy concerns as these were alleviated by the study information sheet.

**Conclusions:**

This study contributes a series of recommendations for future studies wishing to scale our approach and for the design of bespoke mobile interventions to support this population.

## Introduction

### Background

Binge eating is classified as a distinct period during which an individual experiences a subjective loss of control over eating, eats notably more or differently than usual, and feels unable to stop eating or limit the type or amount of food eaten [1]. It is thought to affect approximately 5% of women and 4% of men worldwide in some form over *their life course* [2]. Binge eating is a precursor for, and is symptomatic of, clinical eating disorders, including bulimia nervosa and binge eating disorder [3]. In bulimia nervosa, binge eating is typically followed by the purging of calories from the body in an attempt to counteract a binge eating episode—for example, through vomiting, use of laxatives or diuretics, extreme dieting, or excessive exercise—whereas individuals with binge eating disorder do not engage in purging practices [4]. Binge eating is often a hidden behavior conducted in secret [5] and can lead to extreme feelings of shame, worthlessness, and lack of control, which can have major impacts on an individual’s mental health and emotional well-being [6]. Research has shown that clinical binges (ie, from those with an eating disorder diagnosis) yield the same subjective experiences before and after the binge as those reported from a nonclinical binge eating episode [7].

Anxiety and depression are prevalent in people with binge-eating behaviors [8-10], and research indicates that binge eating is used as a strategy for regulating negative affect (often used as an umbrella term to refer to emotive experiences such as mood, emotion, impulses, and stress response [11]). In addition, as people with binge-eating behaviors are often within the normal to obese BMI category range [12], people in this population report feelings of being unworthy of mental health support and not identifying themselves as having an eating disorder. All this, paired with the secretive nature of binge-eating behaviors, means that the identification and treatment of binge eating are particularly challenging [5].

A growing body of literature has identified opportunities for mobile sensing (ie, the collection and use of data collected from sensors embedded within mobile devices such as smartphones) to detect mental health behaviors such as schizophrenia [13], bipolar disorder [14], and depression [15]. With the exception of the study by Juarascio et al [16], who looked at heart rate variability as a risk predictor for emotional eating episodes, no research has explicitly investigated the potential for mobile sensing in relation to disordered eating behaviors. This is an underexplored area of research, which has significant opportunities for (1) identifying the contextual and situational factors associated with binge-eating episodes and (2) providing improved access to support and behavior prevention through context-aware interventions [17,18].

Our research aimed to contextualize the experiences of people engaging in self-identified episodes of binge eating, with or without subsequent purging activities, by understanding the types of activities that a person might be engaging with in and around the occurrence of an episode. As this was a first-of-its-kind exploratory work, we used a broad range of mobile sensors that already exist in mobile phones (eg, location sensors can indicate whether someone is spending a lot of time at home; movement sensors can give us an idea of how much activity a person has been engaging in; app usage sensors can indicate how much time someone is spending on social media or healthy eating and fitness apps). We asked participants to provide daily self-reports of their mood (collected in both the morning and evening) and to self-report any episodes of binge eating (logged through a button press to capture the time of the episode, with the option of providing further information in the form of free-flowing text). This provided us with a measure of the differences in behavioral features extracted from smartphone data on days with and without incidents of binge eating in an attempt to inform future context-aware mobile interventions to support this population.

We describe the development of the DeMMI (Detecting Mental health behaviors through Mobile Interactions) app, which was refined in consultation with service users. We then describe a 6-week remote study of 20 participants (10 with experiences of binge eating and 10 without any mental health issues, who reported twice daily mood logs and acted as a comparison group). Our contributions from this paper are three-fold: (1) first, we provide a set of reflections around the challenges of conducting work with the binge-eating population and the benefits of remote, anonymous engagement; (2) second, we provide unique insights into the successes and challenges surrounding our mobile sensing approach (from a pilot study perspective) and how this might be better scaled in the future for larger-scale studies over longer periods; and (3) finally, we provide a set of recommendations for the design of future context-aware interventions aimed at supporting people experiencing binge-eating behaviors.

### Use of Ecological Momentary Assessment for Monitoring Mood and Binge Eating

Cross-sectional and longitudinal studies are useful for understanding the long-term risk factors of poor mental health, including those that contribute to binge eating (see the study by Burton and Abbott [19] for review). However, they are much less useful for understanding the more immediate contextual and situational factors that directly contribute to fluctuations in mood that accompany binge-eating behavior. Ecological Momentary Assessment (EMA) techniques are better able to provide information on these contextual factors. EMA involves the recording of problematic moods, thoughts, and behaviors, as well as the events that immediately precede them, to identify predictive patterns. Although early EMA involved the use of paper-based diaries, more recent research has used digital tools (eg, mobile phones and web-based apps) to gather real-time self-reported data. Data collected through digitally enhanced EMA not only have the potential to enhance the understanding of binge eating in research settings but can also be used in therapeutic settings, as well as by individuals, to better understand and monitor individual patterns related to poor mental health. Indeed, a large proportion of smartphone apps specifically for binge eating [20-23], as well as for mental health [24-30], typically involves self-reporting and repeatedly prompting participants over time [31]. Such mobile monitoring apps are typically well-received by young people [32].

EMA relies on the self-report of affective states; however, there is much heterogeneity in the way these affective states are measured. The self-report questions used in smartphone-based EMA are often literal translations of clinical tools [24,26,33,34]. For example, The Positive and Negative Affect Scale (PANAS) involves participants indicating the extent to which they are experiencing 10 types of positive (eg, *alert*) and negative (eg, *upset*) affect using 5-point Likert scales [35]. Although the original PANAS is generally considered too long to be applied with high frequency in EMA [36], subscales and shortened forms have been delivered using smartphone EMA with good response rates, even when used multiple times daily [33]. Furthermore, other approaches have attempted to reduce the burden on participants through the use of visual scales. For example, the Self-Assessment Manikin uses 3 icon-based scales to measure pleasure, arousal, and dominance [37]. In their original form, the icons are abstract outlines of a human-like figure; however, other implementations have also used more realistic representations [26]. Studies have found preferences for these briefer visual scales when compared with more repetitive traditional EMA [26,38].

### Mobile Sensing Approaches for Supporting Mental Health and Binge Eating

The ubiquity and sensing capabilities of smartphones make them attractive tools for the passive collection of multimodal sensor data 24/7. Compared with EMA, they are objective, less burdensome, have a higher temporal resolution, and provide rich data streams to infer aspects of users’ social context and behavior in naturalistic conditions [39,40]. Research has shown the potential of these data to monitor and support mental health conditions, including depression [15,38,41-44], schizophrenia [13,45-48], bipolar disorder [14,49-53], stress [54-58] and anxiety [59-62]. Typically, behavioral features (metrics quantifying aspects of individuals’ routines and activities) are computed from smartphone and wearable data, and their role in tracking, classifying, or predicting events of interest (eg, depressive states and hospital readmission) is explored via analytical methods. Clinical scales, medical records, and patient-reported outcomes are often used as the ground truth to validate models built on top of behavioral features. However, to the best of our knowledge, only Juarascio et al [16] explored mobile sensing in the context of eating episodes associated with negative emotions. They monitored 21 people with clinically significant emotional eating behaviors for 4 weeks using a wrist-worn device. The results showed that time and frequency domain features of heart rate variability could be used to classify 30-minute periods with and without emotional eating episodes better than chance. Although they showcased the importance of wearable data in supporting binge-eating monitoring, patient perspectives on mobile sensing and the role of smartphone phone data remain unexplored.

For eating disorders more broadly, research has shown that smartphone apps could increase patients’ access to treatment [18] because of the anonymity they afford when considering the barriers people face in seeking clinical help (eg, shame and fear of stigma) [5]. Furthermore, in light of near-ubiquitous smartphone use in modern society, these devices are uniquely positioned to support access to resources by promoting help-seeking and self-management behaviors [18,63]. Smartphones can enable personalized monitoring, which can aid in the identification of high-risk situations derived from behavioral and situational contexts extracted from multimodal data. Alongside their capabilities for digital intervention provision, they offer a powerful platform for delivering support at optimal times [17,18]. Currently, there are a number of apps designed primarily for people with disordered eating behaviors that have been studied in the literature, including *Recovery Record* and *RiseUp* [18,64]. Both apps use self-monitoring techniques and provide users with a set of coping strategies to try. In particular, *Recovery Record* uses EMA to facilitate self-monitoring [65,66] and has some features that are similar to the DeMMI app (eg, the ability to track mood and episodes of binge eating). However, this requires a significant amount of active tracking from the user (ie, daily diaries, logging of meals, and the feelings surrounding them), which can be a laborious task. Our study is interested in how passive approaches to monitoring can be leveraged, allowing us to potentially automatically detect contextual or situational triggers for episodes of binge eating. This would ultimately remove some of the tracking burdens from the user and provide indications of where digital interventions might be best positioned to help them. As a first step toward this goal, our study explores the individual differences in smartphone behavioral features between days with and without binge-eating episodes, framed around the experiences and needs of our users, as well as the current and future challenges faced by the mobile sensing community in detecting binge-eating episodes and delivering digital interventions.

## Methods

### Overview

This study was conducted in 2 stages. The first involved the development of the DeMMI app. As part of this, we conducted a consultation session to explore whether the types of sensor data we were proposing to capture were seen as useful and appropriate, as well as to gather feedback on some specific app features relating to self-report. The second stage involved conducting a 6-week period of data collection with 10 participants experiencing binge eating (logging both their mood and episodes of binge eating) and 10 comparison participants (logging only mood).

First, we present the ethical considerations of this study. We then present the 2 stages of research separately, first describing the development of the DeMMI app before moving on to discuss our fieldwork study methods and findings.

### Ethical Considerations

Ethical approval for this work was obtained from York St John, United Kingdom, University Ethics Committee (RECPSY00012), and the study adhered to the British Psychological Society ethical guidelines. The activities for the stage 1 consultation session were constructed collaboratively within the research team, which was made up of a clinical mental health professional and multiple highly experienced researchers with expertise in engaging people with a range of mental health issues in qualitative workshops and interviews. The session was led by one of these experts and supported by an experienced postgraduate student working in the field of disordered eating and self-harm. Both facilitators were careful in creating an open and nonjudgmental space during the sessions. In stage 2, the research team created a safeguarding protocol before the commencement of remote participant recruitment. Participants were fully informed that their data were not being actively monitored and that the research team members were not mental health professionals. However, we conducted weekly well-being checks via WhatsApp or SMS text messaging (depending on participant preference) during the study. These asked participants how they were managing with the study and offered them an opportunity to reach out for support if required. Although we did not have any requests for support during the study, we were prepared to point the participants to local services.

### Phase 1: DeMMI App Development

#### Overview

Given the sensitivity of the data we wanted to collect, as well as an acknowledgment that reporting on disordered eating behaviors might, in itself, be considered a trigger, we first conducted a consultation to (1) gain an understanding of early perceptions toward the data we intended to collect with the app; (2) gather ideas on the best ways of collecting self-reported data in a sensitive way; and (3) understand any specific opinions potential users might have around the rate of data capture, anonymity, and offers of support. We report the main insights drawn from this consultation to provide context for our design decisions before moving on to describe the DeMMI app itself.

#### Consultation Activity

**Figure 1:**
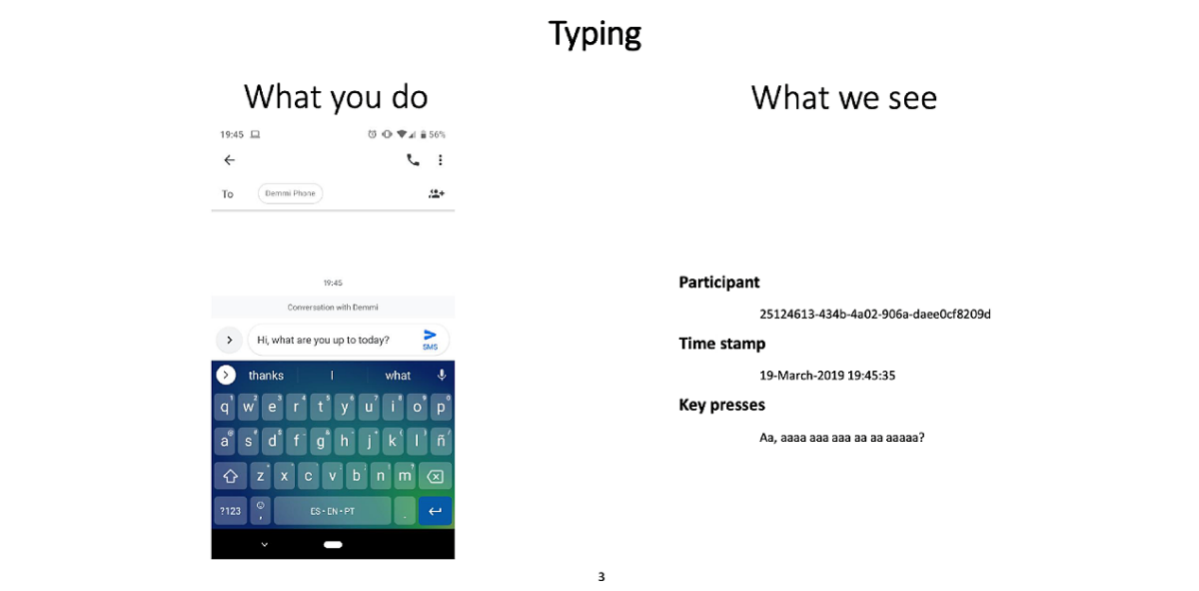
Example of one of our What You Do/What We See slides used to explain to participants what smartphone data we wanted to capture (ie, the participants’ interaction) and exactly what we would see from this interaction. Slides were created to represent the following: app notifications, app use, typing, battery, calls, SMS text messages, data sent or received, screen locks or unlocks, time zone, Wi-Fi nearby devices, Bluetooth nearby devices, ambient light, weather, location, ambient noise, and activity recognition data.

We engaged service users (n=2) from York, United Kingdom, in a consultation session to develop and iterate key design decisions related to the DeMMI app. One of the service users had lived experience of binge eating, and the other had lived experience of self-harm (as a parallel project aimed to explore the feasibility of DeMMI app use among individuals who self-harm). The consultation explored the participants’ perceptions of mobile data collection through both passive and active means. In terms of passive data capture, we explored participants’ general perceptions of mobile sensed data collection and sought specific feedback on the intended data collection proposed within the study—specifically, how they felt about us collecting potentially invasive sensor data. To facilitate this activity, we used a bespoke *What You Do/What We See* resource (Figure 1; the full resource can be viewed in Multimedia Appendix 1), which showed participants the output of every sensor. We discussed each sensor in turn and responded transparently to any questions. In terms of active data capture, we explored participants’ general perceptions of using apps to log mood and behavior, as well as their specific feedback on our proposed app logging mechanisms in the DeMMI app. There were 2 specific features for which we were interested in gaining feedback. First, we were interested in understanding service users’ perspectives on the use of the PANAS (which is widely used in EMA research with the general population [33-35]) to assess mood multiple times each day. Second, we were interested in the use of a 1-click logging mechanism of binge-eating episodes based on the *oops* button developed by Tulu et al [67]. During the session, data were audio recorded to allow the team to listen back to the session; however, as we only had 2 participants, we did not thematically analyze the data. Instead, we took notes during the session and cross-checked all key findings with the participants.

The key findings from the consultation may be summarized as follows:

1. Participants were generally positive about the use of mobile sensing data for research and intervention purposes, identifying multiple beneficial uses, including trigger identification, improved (ie, more accurate and less effortful) behavioral logging, crisis management to prompt positive behaviors, and supported self-reflection. Any concerns they had regarding passively sensed mobile data (eg, *that’s creepy*) were overcome once data privacy had been reassured using the *What You Do/What We See* activity.
2. Discussions of the *oops* feature were mixed and led to modifications. Participants liked the idea of an easily identifiable, user-friendly, and low-effort button to quickly and efficiently record any incidences of problem behavior but felt that labeling this as *Oops* was condescending, whereas *Log* was found to be more amenable. Participants also described how this method of behavior logging could be improved by allowing the optional entry of descriptive text—either at the time of making the log or afterward—so that they could add additional contextual information.
3. Finally, participants expressed concerns regarding the use of the PANAS, both in terms of the content of the scale and the frequency of its proposed use in the study (eg, If I had to do this three times a day, it would be a bit of a trigger for me...Just the word, ashamed, like if I had to go over that three times a day, I probably wouldn’t do it, to be honest”). A simple way of logging mood (eg, using smiley faces) was suggested as a preferable alternative.

#### DeMMI App Development

**Figure 2:**
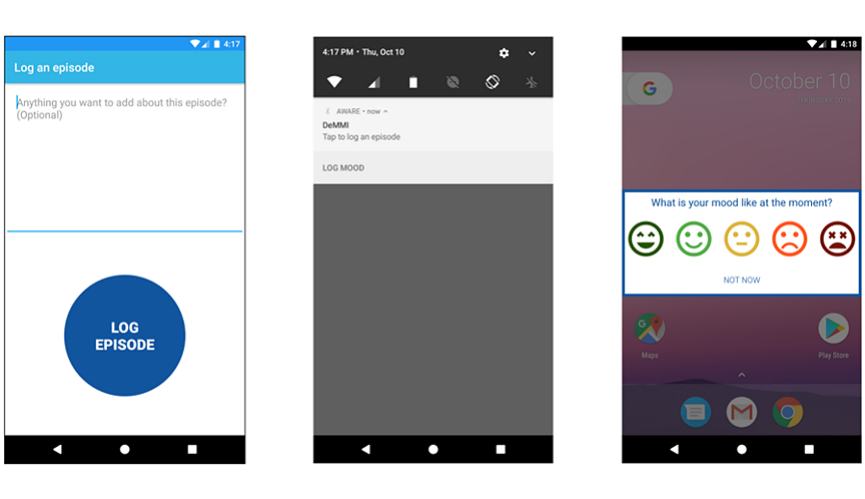
Screenshots of our button to self-report episodes (left), the persistent notification that allowed participants to open the episode and mood reporting screens (middle), and the mood reporting strings with 5 face emojis (right).

The DeMMI app is based on the AWARE [68] client for Android 7.0 or newer, which we used to collect smartphone sensor data 24/7 from 14 sensors. The AWARE framework is an open-source mobile sensing platform used in context-aware mobile computing research. We collected data on accelerometer readings, app use, notifications, battery, Bluetooth, calls, keyboard events, ambient light, locations, SMS text messages, physical activity, screen power events, screen touch events, and Wi-Fi data. On the basis of our consultation, we modified the client to allow participants to log binge-eating episodes using a *Log episode* button placed below a textbox for open-ended feedback related to the episode (see the left screenshot of Figure 2); both were shown after tapping on the main body of a persistent notification labeled “DeMMI. Tap to log an episode.” Participants were also able to log their mood using a scale with emoji faces, ranging in expression and color, to visually represent affective states on a 5-point scale ranging from very positive (represented by a very happy, smiling face) to very negative (represented by a very sad face; see the right screenshot of Figure 2). This scale was automatically shown on screen every day at 9 AM and 9 PM, and as soon as a participant tapped a face, the app logged the choice and hid the instrument. We provided a *Not Now* button so that participants could ignore the prompt and allowed them to report their mood outside the scheduled times by tapping the bottom area of the persistent notification labeled as “LOG MOOD.”

### Phase 2: Fieldwork

#### The COVID-19 Context

This study was conducted with participants located in England, United Kingdom, between June 1 and August 14, 2020, as the COVID-19 lockdown restrictions were gradually eased. Before this point, individuals had not been permitted to leave home except for limited purposes (eg, shopping for essential items, exercise, and medical care), and all nonessential shops, libraries, places of worship, and playgrounds were closed. During the time of our study, changing restrictions permitted individuals from ≥1 household to meet outdoors in groups of 6 while maintaining 2 m social distance (June 1), whereas individuals living alone could form a *support bubble* with 1 other household (June 13). From July 4, the premises reopened with strict social distancing and hygiene measures in place, and gatherings of up to 30 people were allowed both inside and outside private dwellings. Those facing disordered eating behaviors are thought to be particularly vulnerable during the pandemic. A study by Branley-Bell and Talbot [69] found that the COVID-19 pandemic had a profound negative impact on people with eating disorders, whereas Schelgl et al [70] found that 49% of patients reported a deterioration in eating disorder symptoms because of the COVID-19 pandemic, and 47% of binge-eating group patients reported an increase in binge-eating symptoms.

#### Participants

Participants were recruited via several channels, including social media, the regional branch of a UK-based mental health charity (York Mind), and York St John University research participant recruitment forums. The study was advertised as a mobile sensing study for mental health, recruiting participants aged ≥18 years who currently reside in the United Kingdom and have an Android phone as their primary device. Participants were recruited into two categories: (1) those with experiences of binge eating, defined as “Eating an amount of food that you consider excessive, usually very quickly during a single session, eating until you feel uncomfortably full, eating when you’re not hungry, eating alone, eating secretly, and feeling depressed, guilt, ashamed or disgusted after eating. This is often, but not always, accompanied by behaviours to counter the binge-eating (e.g. skipping meals, vomiting, over-exercising)” and (2) those with no current mental health difficulties (comparisons). Participants with experiences of binge eating were not required to have a clinical diagnosis to take part in the study. Participants registered their interest by either directly emailing a designated member of the research team or completing a web-based form. Prospective participants were emailed the study information sheet and consent form and could ask any questions via email or phone call. All participation in the study was conducted remotely. A total of 20 participants took part in the study (n=10 [50%] with experiences of binge eating and 10 [50%] with no history of mental health issues). All participants were aged between 18 and 36 (mean 25) years and were almost exclusively female (with 1 male in each group).

#### Study Methods

Once recruited into the study, the participants were sent an onboarding pack via email. This included a link to the DeMMI app Android Application Package (an Android package file format allowing the app to be downloaded via the link) and a set of step-by-step instructions for downloading, opening, optimizing battery life, and setting permissions on the app (Multimedia Appendix 2). Instructions on how to join the study with a unique identifier were also provided, as were instructions on how to log mood, log episodes, and uninstall the app.

Participants were asked to run DeMMI for a total of 6 weeks but were instructed that they could uninstall the app at any time, and following removal, all collected data would be deleted from their phone. All participants were asked to log their mood twice a day (at 9 AM and 9 PM), and the binge-eating group was asked to log any episodes of binge eating using the *Log episode* button, with the option to provide free-text information regarding the episode. To enhance engagement in the study and ensure that safeguarding protocols were being followed, participants were contacted once a week via SMS text messages to flag any potential problems. Following the study, participants were given the opportunity to take part in an optional interview to discuss their experiences during the study and provide any feedback regarding how we might improve the app functionality in the future. The interviews were conducted via telephone or video call (depending on participants’ preferences).

### Data Analysis

#### Quantitative

Smartphone behavioral feature analysis was conducted on the data collected from the binge-eating group, with comparisons across days that an episode had been reported and days that an episode had not been reported. We used the Reproducible Analysis Pipeline for Data Streams (RAPIDS) [71,72] to preprocess, clean, and extract behavioral features from the smartphone data collected with the AWARE framework. RAPIDS is a reproducible pipeline that allows for the processing of mobile sensing data. According to our protocol, every participant had to be monitored for 42 days (ie, 6 weeks); however, in practice, their smartphones could run out of battery, our sensing app could crash, or it could have issues synchronizing the data. Therefore, we expected some of these days to be missing all, most, or some of the data. We measured the quality of our smartphone data through the concept of valid sensed days; we labeled a sensed day as valid if we had 8 hours of data with at least 30 sensed minutes each. A sensed minute is a 60-second window with at least one row of data from any smartphone sensor.

Once the data were processed, we used the Nonoverlap of All Pairs (NAP) index [73] to measure the probability that a behavioral feature value drawn at random from any episode day would exceed that of a feature value drawn at random from any nonepisode day. The NAP analysis provided an indication of effect size and offered directions for future work that might use such sensor-driven approaches in the context of binge eating.

#### Qualitative

All free-text episode logs collected during the study were collated and subjected to content analysis [74] to explore any recurrent themes of discussion across participants that might provide future directions for focus. Our analysis involved assigning codes to the lines of the data and grouping them into themes. The interviews were all audio recorded and transcribed verbatim. Interview transcripts were thematically analyzed using a deductive approach that saw codes created at the paragraph and sentence level [75].

## Results

### Overview

In this section, we describe and summarize the smartphone data, mood survey notifications, mood scores, and collected binge-eating episode data. To explore the relationship between smartphone data and binge-eating episodes, we computed 12 behavioral features related to social interaction and physical activity across location, Bluetooth, physical activity, and screen sensor data and analyzed the difference in their values between days with and without self-reported binge-eating episodes. We have shared the code of our mobile app and analysis pipeline to enable the reproduction of our methods [76,77].

We present our analysis in 3 parts. We first look at the entire cohort of data for all 20 participants (both the comparison group and the binge-eating group) and provide an outline of the valid sensed days that we were able to collect (providing an indication of how long participants in both groups engaged in the study before deleting the app and how well the software functioned in relation to collecting the sensor data). Within this cohort level data, we also provide a comparison of mood scores across all 20 participants to explore whether there was any difference between mood reporting across the 2 groups. In the second part, we look specifically at the binge-eating data and the differences in sensor data collected on days when an episode was reported versus days when an episode was not reported. We first provide a content analysis of binge-eating episodes and then discuss the NAP analysis conducted. Finally, we report the interview data across both groups, focusing on the usability and acceptability of our approach and the app itself.

### Part 1: Entire Cohort Data

#### Valid Sensed Days Across All Participants

Participants were asked to keep the app running on their phones for 6 weeks (approximately 42 days). In relation to study engagement, the 2 groups were relatively similar; the binge-eating group had a total of 395 total days with the app, with 20% (2/10) deleting the app before the end of the study (P02BE after 15 days; P06BE after 36 days). The comparison group had a total of 397 days with the app, with 10% (1/10) of participants deleting the app before the end of the study (P13C after 5 days). There were some differences in the valid sensed days; however, the binge-eating group had 72.4% (286/395) of valid sensed days compared with the comparison group, which had 61.7% (245/397) of valid sensed days. See Table 1 for a full breakdown of the data.

**Table 1:** Participants in the binge eating (N=395 days) and comparison groups (N=397 days) along with the number of days they were monitored and the number of valid sensed days.

Participant ID	Group	Days with the app, N	Valid sensed days, n (%)
P01BE	Binge eating	45	0 (0)
P02BE	Binge eating	15	12 (80)
P03BE	Binge eating	44	42 (95)
P04BE	Binge eating	43	31 (72)
P05BE	Binge eating	41	38 (93)
P06BE	Binge eating	36	6 (17)
P07BE	Binge eating	43	38 (88)
P08BE	Binge eating	43	39 (91)
P09BE	Binge eating	43	41 (95)
P10BE	Binge eating	43	40 (93)
P11C	Comparison	44	31 (70)
P12C	Comparison	44	35 (80)
P13C	Comparison	5	0 (0)
P14C	Comparison	43	40 (93)
P15C	Comparison	44	9 (20)
P16C	Comparison	44	20 (45)
P17C	Comparison	43	42 (98)
P18C	Comparison	43	2 (5)
P19C	Comparison	44	27 (61)
P20C	Comparison	43	39 (91)

#### Mood Logs Across All Participants

Mood logs were collected twice daily at 9 AM and 9 PM. Overall, the binge-eating group logged their mood when prompted by our app notifications with a log rate of 87.1% (548/629 times). The comparison group logged their mood at a higher rate of 90.4% (638/706 times).

However, as seen in Table 2, the binge-eating group had lower overall positive mood scores and was more likely to score their mood as negative (64/596, 10.7% compared with 17/671, 2.5% in the comparison group) or very negative (19/596, 3.2% compared with 0.4%, 3/671 in the comparison group). The binge-eating group was also more likely to report a lower mood log in the mornings (34/298, 11.4%) and evenings (39/298, 13.1%), reporting negative or very negative scores compared with comparisons (6/330, 1.82%, and 14/330, 4.2%).

**Table 2:** Count of mood score categories from 0 (very positive) to 4 (very negative) for the comparison and binge-eating groups.

Mood Score	Binge-Eating Group	Comparison Group
0 Very Positive	34	52
1 Positive	335	453
2 Neutral	144	146
3 Negative	64	17
4 Very Negative	19	3

We computed the rate between the number of mood surveys displayed on the screen and the number of surveys that should have been triggered (2 per sensed day). Our participants had a mean trigger rate of 92.9% (986/1062; SD 10, minimum 59.4, maximum 100), which shows our surveys were delivered reliably. We also calculated the rate between the number of surveys that were answered and the surveys displayed on the screen. We obtained an average answer rate of 88.4 (SD 6.71, minimum 67.5, maximum 96.2), suggesting that our mood survey instrument was easy to use, which was further confirmed by the fact that the number of ignored surveys was on average 1.3 per week throughout the study’s 6 weeks. We also gave participants the opportunity to report their moods outside the scheduled time. This functionality was rarely used, with a total of 95 self-initiated mood reports and an average of 5 responses (SD 5.9, minimum 0, maximum 18) across all participants. However, we highlight that 26% (25/95) of these surveys were used to correct a previous self-reported mood score (defined as any new report made within 2 minutes of the previous one).

### Part 2: Binge-Eating Data

#### Episode Logs

We had 98 episodes reported across our 10 participants in the binge-eating group (Table 3). Most logs occurred in the evening between 6 PM and midnight (41/98, 42%) or in the afternoon between noon and 6 PM (35/98, 36%), with a smaller number occurring in the morning between 6 AM and noon (14/98, 14%) and a very small number occurring late at night between midnight and 6 AM (8/98, 8%).

**Table 2:** The total number of episode logs (N=98) and episodes logged with additional text for all binge-eating group participants.

Participant ID	Episode logs, n (%)	Episodes logged with additional text (n=62), n (%)
P01BE	6 (6)	0 (0)
P02BE	3 (3)	0 (0)
P03BE	9 (9)	3 (5)
P04BE	6 (6)	2 (3)
P05BE	8 (8)	7 (11)
P06BE	23 (23)	23 (37)
P07BE	19 (19)	18 (29)
P08BE	16 (16)	2 (3)
P09BE	6 (6)	5 (8)
P10BE	2 (2)	2 (3)

Of the 98 episode logs, 36 (37%) were recorded without any additional text. We conducted a content analysis of the remaining 63% (62/98) of self-reported episodes with additional text to explore any related themes of reporting that cut across participants. It should be noted that some episodes involved multiple themes. There were six overarching themes identified from our content analysis: (1) perceived lack of control, (2) emotions, (3) invasive negative thoughts, (4) disordered eating behavior explicitly reported, (5) situational influence, and (6) behavior avoided.

We had 36 explicit reports of binge-eating behavior across participants:

I was watching movies and binged snacks even though i’m not hungry

P05BE

Made coffee muffins when partner was out. Ate 6 in a row.

P06BE

massively over-snacked after not eating much lunch at 12pm

P08BE

There were 17% (6/36) of reports that displayed a perceived lack of control:

tried really hard to not binge, but couldn’t stop myself

P03BE

I’m on my own and I can’t sleep, and I can’t stop snacking

P05BE

Invasive negative thoughts were also explicitly reported 33% (12/36) times: “Self-critical thoughts. Strong emotions” (P04BE); “feel like failure” (P06BE); and “I’m having really bad intrusive thoughts :(” (P09BE). Moreover, participants reported on their feelings and emotions after a binge-eating episode: “feeling quite ashamed now” (P03BE), “feel useless and angry,” and “Feel disappointed and dumb” (P06BE). Participants also reported 15 situational influences, which led them to disordered eating behaviors:

Had to do a job interview today but didn’t go to plan

P03BE

jeans I had ordered arrived today. They didn’t fit and all I wanted to do was sit and cry and eat but I couldn’t because I wasn’t alone

P05BE

every time I see an ad for that massive popcorn chicken from KFC I wanna binge eat the entire thing

P06BE

Finally, there were 17% (6/36) of remaining reports related to disordered eating behaviors that were avoided, despite the thought processes around binge eating being there, because of factors such as not being alone or because of the acknowledgment of the negative effects of the disordered eating behavior:

want to make myself sick in the bathrooms but it’s empty and I’m scared my partner will hear.

P06BE

didn’t realise how much I was eating till I finished[...] but I am trying to stop purging as it is starting to have negative physical effects

P03BE

In the case of P06BE, there were several reporting incidents that were framed positively (5/36, 14%):

Feel good didn’t have cake etc at cafe as I would 100% scoff and eat too much and feel ill like i usually do

P06BE

This may have been perceived as a restraint in relation to the avoidance of a binge-eating episode; however, it is worth noting that the deliberate restriction of food can also be problematic and a prelude to binging.

#### Smartphone Data on Episode Versus Nonepisode Days

P01BE had only 2% (1/45) valid days, P02BE had 93% (14/15) of valid days, and P06BE had only 17% (6/36) valid days; as such, they were excluded from our behavioral feature analysis. This left us with the remaining 70% (7/10) of participants. We explored the difference in magnitude between days when our participants did and did not report an episode in relation to two self-reported mood scores (those logged in the morning between 6 AM and noon and those logged in the evening between 6 PM and midnight), as well as 12 behavioral smartphone features extracted in daily segments that span midnight to midnight. We included the following smartphone features: geographical location variance, total distance traveled, radius of gyration, time at home, stationary (to moving) ratio, the total stationary time, number of distinct Bluetooth devices sensed around the phone, number of screen unlocks, total screen time, time of first screen use, total number of mobile apps used, and the entropy of all mobile apps used (a wider variety of apps produce a higher entropy value). The sensors that provide these features, a layman’s description, and the implementation of these features can be found in the RAPIDS documentation [71].

We framed our problem as 7 n-of-1, or single-case, experiments with alternating AB phases. A phase is formed by consecutive days without binge-eating episodes, whereas a B phase is formed by consecutive days with binge-eating episodes. Overall, 82% (36/44) of the B phases across all participants were 1 day long (mean 1.3, range 1-4 days). The A phase across all participants (N=48) was, on average, 5 days long (range 1-28). Table 4 shows the breakdown of the average phase length and range for each participant.

**Table 3:** Average length (days) of phase A (no episode; N=48) and phase B (episode; N=44).

Participant ID	A phases, n (%)	A phases length, mean (range)	B phases, n (%)	B phases length, mean (range)
P03BE	7 (15)	5.1 (1-10)	6 (14)	1.3 (1-2)
P04BE	6 (13)	6.3 (1-21)	5 (11)	1 (1)
P05BE	6 (13)	5.5 (1-13)	6 (14)	1.3 (1-3)
P07BE	9 (19)	3.1 (1-9)	9 (20)	1.7 (1-4)
P08BE	11 (23)	2.6 (1-7)	11 (25)	1.3 (1-3)
P09BE	6 (13)	6.3 (1-14)	5 (11)	1 (1)
P10BE	3 (6)	13.7 (1-28)	2 (5)	1 (1)

We used the NAP index [73] to measure the probability that a behavioral feature value drawn at random from any phase B will exceed that of a feature value drawn at random from any phase A. We used the R implementation provided by Pustejovsky et al [78], concatenating all (1–N)A phases into a single A phase and all (1–M)B phases into a single B phase and setting a confidence threshold of 0.95 and an expected direction of improvement given by the sign of the standardized mean difference of A and B values ([mean_B_ – mean_A_]/SD_[A,B]_).

In Table 5, we share the behavioral features of each participant that had a NAP medium effect (an index between 0.66 and 0.92) or a NAP strong effect (an index between 0.93 and 1) [73]. Only the strong effects belonging to P10BE were statistically significant after adjusting for multiple tests within participants using the Benjamini and Hochberg method [79]. P04BE, P05BE, P07BE, and P10BE self-reported feeling worse on days when they binge ate; P04BE had morning mood at a medium effect; P05BE and P07BE had evening mood at a medium effect; and P10BE had evening mood at a strong effect. P05BE, P08BE, and P09BE had screen features with a medium effect (P08BE and P09BE used or unlocked their phones less on days when they binge ate). P03BE had app entropy with a medium effect (he or she used a wider variety of apps on days when they binge ate). P04BE and P10BE had time at home with a medium effect (they spent more time at home when they binge ate). P04BE, P05BE, and P10BE had a medium and strong effect for total distance (P04BE traveled more and the others 2 less in days when they binge ate). P09BE and P10BE had a stationary time and ratio with a medium effect (they moved around less when they binge ate). Finally, P10BE had location variance and radius of gyration with a strong effect (he or she traveled less when they binge ate) and unique Bluetooth devices with a medium effect (he or she was arguably around fewer people or public places when they binge ate).

**Table 4:** Smartphone features that showed a medium or high NAP^a^ effect between phases A and B of participants that binge eat.

Participant ID	Feature	Standardized mean difference	NAP (SE)	NAP, 95% CI	NAP effect	Adjusted *P* value
P03BE	App entropy	0.67	0.69 (0.1)	0.46-0.84	Medium	.45
P04BE	Time at home	0.64	0.73 (0.9)	0.46-0.89	Medium	.45
P04BE	Mood morning	0.62	0.68 (0.1)	0.41-0.86	Medium	.45
P04BE	Total distance	0.38	0.67 (0.16)	0.40-0.85	Medium	.45
P05BE	Total distance	−0.58	0.73 (0.11)	0.51-0.87	Medium	.29
P05BE	Mood evening	0.79	0.69 (0.13)	0.47-0.85	Medium	.29
P05BE	Screen unlocks	0.36	0.67 (0.08)	0.45-0.83	Medium	.33
P07BE	Mood evening	0.70	0.68 (0.08)	0.50-0.81	Medium	.25
P08BE	Screen unlocks	−0.63	0.69 (0.08)	0.51-0.83	Medium	.29
P09BE	Stationary time	0.79	0.68 (0.16)	0.41-0.86	Medium	.44
P09BE	Screen time	−0.81	0.67 (0.16)	0.41-0.85	Medium	.44
P10BE	Mood evening	1.67	0.99 (0.02)	0.58-1.00	Strong	.046
P10BE	Radius of gyration	−0.46	0.98 (0.02)	0.57-1.00	Strong	.046
P10BE	Location variance	−0.32	0.98 (0.02)	0.57-1.0	Strong	.046
P10BE	Total distance	−0.68	0.98 (0.02)	0.57-1.0	Strong	.046
P10BE	Stationary ratio	1.47	0.91 (0.09)	0.5-0.99	Medium	.07
P10BE	Time at home	1.01	0.88 (0.06)	0.46-0.98	Medium	.09
P10BE	Bluetooth devices	−0.88	0.83 (0.07)	0.42-0.97	Medium	.13

^a^NAP: Nonoverlap of All Pairs.

### Part 3: Poststudy Interviews

Poststudy interviews were optional. Among the 10 binge-eating group participants, 3 (30%) consented to the poststudy interview; among the 10 comparison participants, 5 (50%) provided consent. Of the 20 participants, this provided a total of 8 (40%) interviews for analysis. Interviews lasted between 14 and 42 minutes each, were conducted via telephone, and were audio transcribed for later deductive thematic analysis. A total of 45 codes were initially created and then further grouped into themes. There were four overarching themes identified: (1) positive and negative impacts of lockdown, (2) phone habits, (3) mood and episode logging, and (4) the usability of the DeMMI app.

#### Positive and Negative Impact of Lockdown

In the interviews, participants were asked how their moods might have changed during the lockdown. Most participants discussed their mood negatively, with both groups reporting fluctuations in their mood during the lockdown and possibly triggering episodes for those in the binge-eating group:

I was doing a lot worse when lockdown got really bad.

P09BE

it was very much up and down...

P13C

I definitely think it’s been a lot more up and down...as soon as something tiny goes wrong...I would have like a full-on breakdown. Then I’ll comfort eat, have a binge or start like picking at myself

P07BE

Several recurring themes were noticed in the interviews, which were related to the negative impacts of the lockdown on one’s mood. Some participants highlighted feelings of hopelessness and that the uncertainty of the situation was causing stress and anxiety:

It can feel quite hopeless as we don’t know when it will change

P10BE

I think like everyone, it’s had a negative impact...there was that sort of novelty factor and we weren’t quite sure how long it was gonna last.

P11C

it was definitely a bit more scary, and because we didn’t know how long it was gonna go on for. A bit unsettling...that was a bit stressful

P15C

However, there were also some positives reported by both groups. Approximately 25% (2/8) of participants mentioned how the lockdown had encouraged them to have better time management, as the time usually spent getting ready and commuting to work could be used to spend more time with people around them and participating in activities that they enjoyed, such as exercising and volunteering:

there’s been a lot of benefits of lockdown...I managed to get into a nice little rhythm once my routine kinda reset

P15C

it’s [lockdown] given everyone a focus which in some ways has helped my social anxiety, I’ve been volunteering which I wasn’t before

P10BE

Furthermore, although some participants highlighted that being able to exercise “makes [them] feel good and it kinda like refreshes and resets [them]” (P1C), for members of the binge-eating group “not being able to go and do exercise as much, just feeling very tired all day” (P07BE) was often seen as a cause of stress that could trigger a vicious cycle of binge-eating episodes and dietary restrictions. Despite this, the same binge-eating group participant reported how having regular face-to-face social interactions during and after the lockdown led to a positive impact on their mood:

if I go into placement. I work well, it’s a good day, but if I’m at home it’s not a good day

P07BE

However, with lockdown limiting face-to-face contact, participants in this group could be at an even higher risk of triggering a binge-eating episode.

#### Phone Habits

Long periods of staying at home during a pandemic, with limited knowledge of the virus, were seen to take a toll on participants’ mental health, and with restrictions on socializing, participants discussed beginning to form new habits, especially with their phone and social media use. During the lockdown, most of the participants reported a noticeable increase in their phone use:

I’ve been on my phone a lot since lockdown started.

P09BE

I definitely use my phone a lot more than I used to...I wouldn’t have done as much if I didn’t have as much free time as I do now.

P15C

I would say that I was probably spending a bit more screen time in lockdown...Not having that structure.

P14C

A participant clarified that this was not because of taking part in the study:

I think it’s more lockdown that’s increased my phone behaviours not really the app.

P07BE

The major causes for this change in phone use behaviors noted by many participants were procrastination and boredom, particularly as the lockdown period was during the summer break for university students and/or some participants were forced to take a break from work:

A lot more procrastination, just not really using it for anything useful.

P07BE

I’m into the habit when I’m not doing anything, I’m much more procrastinating on my phone than I used to be.

P09BE

I was using that a lot more, and maybe even out of boredom.

P13C

maybe out of boredom a little bit and maybe just out of habit as well.

P14C

A few participants also discussed the negative impact of social media and phone use on one’s mental health:

the increase in my phone usage probably contributed to the decline in my mental health.

P10BE

when I’m on social media and I see negative things. So, when I get news alerts it’s always negative...I think Twitter was the worst for me...when everything was going on with coronavirus and like the Black Lives Matter movements...everything really made me feel down and depressed.

P17C

Moreover, 60% (3/5) of the comparison group participants discussed the positive impact of using the DeMMI app:

it made me think about my use of social media or being on my phone and if I thought that correlated with my mood...I was thinking about how work affected my mood, rather than my usage of my phone.

P14C

I did think about phone usage as well because when I was thinking about what the app is sort of looking at, I would reflect and think that actually I probably use my phone a lot more than I thought I did.

P13C

I noticed how often I was using my phone, and I was quite conscious of it in the first couple of days, I did notice patterns in my mood too.

P15C

This seemingly led some participants to reflect on how their phone use correlated with their mood.

#### Mood and Episode Logging

The binge-eating group participants were asked to expand on what they felt triggered them to experience an episode and how they classified an episode during the study (it should be noted that the comparison group was only instructed to log their mood). For one participant, the impact of social relationships on the occurrence of episodes was noted, and the passing of a close family member had affected their mood:

in terms of the binge eating...Cause, my boyfriend at the time...we had some sort of issues

P07BE

probably about 6 weeks ago there was a big trigger...my uncle had been ill for a while...And then he died about 6 weeks ago

P07BE

Other binge-eating group participants noted that “intrusive thoughts” (P09BE) and “the increase in my phone usage probably contributed to the decline in my mental health” (P10BE), which might trigger episodes of binge eating. In the interviews, participants were also asked whether they noticed any patterns in their behavior. Approximately 67% (2/3) of the binge-eating group participants also noticed that their mood had improved with the return of social interactions and having more occupied time:

So now it’s a lot less time for overthinking and worrying about things, and more time to actually just be doing stuff.

P07BE

None of the participants in the comparison group reported any concerns with mood logging. On the other hand, one of the binge-eating group participants found it invasive at times because of logging in a public environment:

Sometimes it was annoying because I wanted to look at my phone for something else. Sometimes if I was with someone, I didn’t want them to see what I was clicking so I didn’t do it.

P10BE

However, another binge-eating group participant noted that the app provided them with a level of accountability over binge-eating episodes:

having to log it and recording my episodes has actually been really helpful as like a deterrent, especially for my binge eating. Because it was giving me some accountability for doing it and if I do binge eat then I have to record it. I actually think that it made my like threshold for having an episode a little bit higher.

P07BE

#### Usability of the DeMMI App

When discussing the usability of the DeMMI app, participants mostly provided positive feedback for the app. Many participants commented that it was “easy to use” (P13C, P14C, P15C, P17C, and P10BE). Some of the participants elaborated on the user-friendly aspects of the app:

I liked [how] you could just click on the notification bar and then just tap to add episode

P07BE

I liked how it popped up with the pop up telling you to log your mood now. Because otherwise 100% I would have forgotten.

P09BE

The main issue reported by the participants was related to the app crashing. Approximately 25% (2/8) of the participants commented the following:

I had a lot of issues with the app force closing and not knowing if it was still running, I don’t think in the end I could enable all the features of it.

P07BE

The only thing that did happen, was occasionally when I was browsing the web, so not really when I was using any apps, it would occasionally say the app wasn’t responding.

P15C

Participants provided several specific examples of feedback that could be used to improve the app in the future. 50% (4/8) of participants proposed an option for additional mood logs; a participant suggested the following:

Maybe one around lunch time and one around evening maybe?

P09BE

Another elaborated with a similar suggestion, saying the following:

Because there was lots of days where in the morning and evening I would put a neutral or a happy face, then in the middle I would dip to close to having an episode but not quite have an episode.

P07BE

P10BE commented that they would like the ability to adjust the time of the mood logs:

I am often only just up by 9am or still asleep, so checking in later for a mood score would have been better...sometimes I needed to urgently look at something on my phone, so I clicked off it quickly without thinking and missed logging it.

All of the binge-eating group participants could see the benefits of a future app version to identify or predict a potential episode, as well as the addition of mindfulness and relaxation exercises, safe practices, or positive affirmations:

even it’s just recognising you’re about to have an episode...I think that might in itself even be a bit useful. Like giving the accountability before and after.

P07BE

if I pre-put in some like songs or something that I like and then it would identify it and then prompt you to play the song, rather than needing to go and think about oh I’m going to put on some relaxing music. Or maybe some exercise which uses your mind a bit to distract you.

P07BE

Maybe if you could tailor them to what you find useful. I wouldn’t like an app to tell me what to do. Maybe if you’d set up a reminder to do mindfulness or something.

P10BE

like positive affirmations...Like, a flowchart on what to do.

P09BE

Finally, the participants were asked about privacy concerns regarding automated data collection by the app. Most participants voiced no privacy concerns:

I kinda trusted the study

P13C

Moreover, 25% (2/8) of participants explained as follows:

but if this was just another app on the app store for sure I would be having privacy concerns.

P09BE

no [concerns] because I was told you could see what apps and stuff were open, but you couldn’t see any messages. I was worried initially when I was signing up to the study, but after I read everything and emailed you it made me feel a bit better about it.

P17C

Further open questions were asked surrounding the clarity of the information sheet that was distributed to the participants. Most participants were satisfied with the level of information that was provided to them to understand the purpose of the study and how their data would be collected and handled:

I didn’t have any concerns or anything about that...I think it was very clearly set out, I don’t think anything stood out particularly.

P14C

No [concerns], I was really happy with it...in terms of privacy there was nothing that really stood out in the information sheet that I was, ooh I’m not sure about that or I don’t understand that...it was all pretty straight forward.

P14C

Several participants further highlighted the importance of explicitly discussing data privacy and security within the study:

the information sheet where they were pointing out the privacy settings, I think that stood out the most to me. Because that was the information that I wanted to know the most before I started the study.

P17C

I did at first, just in terms of there’s so much data that you can really get passively from someone’s phone...But I knew what was being done with the data, so I wasn’t too concerned about it.

P15C

## Discussion

### Principal Findings

Our preliminary quantitative results suggest that every participant had various smartphone features that were meaningfully different between days with and without binge-eating episodes. This, in turn, could encourage researchers to investigate fully data-driven approaches to find *hidden* links between smartphone behavioral features and these episodes either via interpretable or black box predictive approaches. However, our qualitative work paints a more nuanced image of the research needed to deliver effective, safe, and ethical digital interventions.

### Perceived Episode Indicators

In the feedback attached to the self-reported episodes, participants described a variety of affective states, comorbid mental health disorders, social interactions, and daily life experiences that either preceded or happened during their binge-eating episode. These findings correspond with the existing literature surrounding binge-eating episodes, which has also identified these factors as potential antecedents of binge-eating episodes [80]. We refer to these as indicators because of their predictive potential and are keen to emphasize that indicators are not necessarily causal contributors to binge-eating episodes. It may be that they are provoked by the latent trigger itself; for example, calling a relative for support may co-occur with binge eating but might not necessarily cause a binge eating episode. Keeping in mind that the basic premise behind a digital intervention based on mobile data is to find behaviors that can be measured to deliver treatment before, during, or after a binge-eating episode, it is crucial to understand and catalog these indicators independently of their causal role.

The idiosyncratic nature of the indicators identified in this study warrants further examination in the form of a longitudinal observational study, which can help clarify the relevance, scalability, and focus of quantifying indicators using mobile data (in our case, screen unlock time, app entropy, time at home, stationary-to-location ratio, total distance, stationary time, screen unlocks, evening mood score, and time of the first screen unlock). For example, a scenario where most of these indicators are related to affective states or mental health disorders such as depression would support the idea of leveraging previous works on general or personalized mobile monitoring of these constructs [81]. Under a different scenario, if most of these indicators are related to situational influences such as social interactions with certain people, work activities, or leisure activities, then it is likely that models to detect these events will have to be highly personalized, given the differences in people’s routines. For example, researchers might have to monitor digital communication between participants and specific relatives or friends or adapt to people’s work, school, or leisure settings (monitoring sleep patterns, drinking patterns, and physical activity). In practice, there might not be clear-cut lines among affect, mental health, and situational influences, as the latter is likely to affect the former, and a participant could report episodes around both types of indicators; however, some might be easier to quantify using smartphone or wearable data (see the *Computation Amenability of Indicators* section).

It is also worth noting that the idiosyncratic nature of the indicators we identified might reflect the idiosyncratic nature of binge eating more broadly. In this study, we defined binge eating in inclusive terms, did not state that it must form part of a specific diagnosis (eg, bulimia nervosa and binge-eating disorder), did not exclude binge eating that is comorbid with other mental health conditions (eg, anxiety), and did not specify whether binge eating must occur in the presence or absence of purging. Previous EMA research suggests that different factors may be more or less important to different clusters of participants. For example, a review by Dingemans et al [82] found differences in the affective dynamics associated with binge eating with purging compared with binge eating without purging. Thus, the previously described work on binge-eating indicators would benefit from collecting this information to understand whether there are commonalities among certain participant clusters.

### Frequency and Life Span of Episode Indicators

We need to understand how often and for how long these indicators happen such that researchers focus their efforts on the most common ones for a participant or a cluster of participants. It is possible that the time a person is exposed to or experiences an indicator will vary and that their relevance fluctuates over time. The former means that the timescale at which mobile data are analyzed will depend on the indicator (eg, should we look at anxiety levels in days or hours in the past?), and the latter implies that models will have to adapt over time to changes in people’s routines and personal circumstances (eg, if being alone triggers binge-eating episodes, what happens when a young adult moves to live on their own after sharing a house with other people during university?). Further compacting this issue, research examining binge-eating episodes has suggested that they may not necessarily be discrete events. More specifically, Wilson and Sysko [83] discuss how binge eating may be best thought of in terms of binge-eating days rather than binge-eating episodes. This is especially important when binge eating occurs without purge behavior, as purge behavior is often considered a clear indicator that an episode has finished [84].

### Severity and Impact of the Episodes Around Certain Indicators

Paying particular attention to the situational influence indicators (eg, being alone or having an argument with a loved one), it is unclear (1) how often they are a proxy for binge-eating episodes (and their likelihood of triggering a false positive intervention); (2) whether the severity of the episodes they pinpoint is similar, as measured by objective means (such as the amount of consumed food, its nutritional value, or the duration of the binge) or subjective means (such as the extent to which they experience a lack of control); and (3) whether the psychological and physical impact of such episodes is similar across episodes (eg, people might not always engage in purging behaviors after an episode). Researchers and patients might prefer to investigate, quantify, and monitor the indicators that pinpoint episodes with the most negative effects. Our lightweight approach to episode logging was well-received by the participants, who provided additional text in 63% (62/98) of the cases. Leveraging natural language processing approaches to gain a better understanding of specific indicator types and their severity of impact on the person is a promising direction for future work.

### Computation Amenability of Indicators

We expect to be able to quantify and detect each indicator to a different degree using smartphone or wearable data. In our study, we computed smartphone features that measured constructs we considered to be roughly related to the indicators reported by our participants. However, once researchers have a better idea of the breadth and depth of indicators in a population, they can decide which behavioral features might be more relevant and need to be extracted. For example, it might be very difficult to measure or anticipate the effect of daily activities such as shopping on body image (P03BE); however, if it turns out that a considerable number of binge-eating episodes occur around body image issues, then we could focus on measuring the affective state induced by them. Alternatively, research has indicated that some individuals may engage with certain apps (eg, calorie-tracking apps) in different ways around binge-eating episodes [1]. Similarly, certain episodes could happen around indicators related to physical activity, sleep disorders, or communication patterns that previous research has had positive results quantifying using smartphones and wearables [47,59,85-87], which are a more direct measurement of the observed phenomena compared with affect and psychological constructs.

### Intervention Candidates for Episode Indicators

Once a relevant indicator is identified and can be reliably measured, the next question is how to intervene to try to prevent binge-eating episodes from occurring and/or provide support for its duration. There is a pressing need for effective binge-eating interventions. The currently available treatments are only effective for up to 50% of individuals with binge-eating disorders and 30% of individuals with bulimia nervosa [88,89]. Of these treatments, the most commonly prescribed is cognitive behavioral therapy (CBT), which involves restructuring an individual’s thoughts, feelings, and behaviors to support more productive outcomes and reduce binge-eating occurrences [90]. Although CBT is typically delivered in therapeutic settings, research has begun to consider its potential when delivered remotely through smartphones (akin to a self-help tool) [17]. Initial research in this space suggests that smartphone-enhanced CBT can be as effective as therapist-led CBT and may be even more effective in attaining some outcomes, such as meal adherence [22]. Such smartphone-based approaches may also be useful at the subclinical level, where individuals binge eat and experience significant distress without a diagnosis.

There are several types of interventions that may be appropriate for in situ delivery in response to the mobile sensed occurrence of an indicator. These interventions can be loosely categorized as (1) prompting and (2) self-management, both of which may be compatible with the CBT framework. Prompting interventions refer to those that are aimed at nudging participants to change their behavior, prompting self-reflection that helps put things in perspective, or even making them consciously aware that an indicator that is typically associated with their binge-eating behavior has been detected. Self-management refers to interventions that support participants by providing access to web-based tools that foster positive mental or physical health. These web-based tools could take many forms, including the automatic recommendation of activities aimed at de-escalating the situation (such as distraction activities available in apps like *Calm Harm* or *Recovery Record*); support messages that are meaningful for the individual; or open communication channels to family, friends, or health care providers that the participant agrees to. Indeed, our participants provided similar recommendations for activities that might support them in future app iterations during the poststudy interviews. Our empirical program suggests that such interventions would need to be personalized to the participant, consistent with previous work on the design of smartphone-based interventions to support mental health [91]. This personalization could be achieved with the support of a therapist (ie, a therapist-mediated intervention) or be self-led (ie, a self-help approach).

### The Risks, Cost, and Effectiveness of an Intervention

Researchers must systematically consider the cost-effectiveness of prompting, self-management, and other types of interventions. This type of analysis has been conducted for HIV [92], physical activity [93], smoking [94], alcohol consumption [95], and CBT-guided self-help interventions for binge eating [96] and should take into account the time and expertise that these kinds of digital interventions would demand from participants and their health care providers [17]. In addition, researchers need to be aware of the risks of delivering an intervention when it is not needed, failing to deliver an intervention when previous deliveries have been successful and participants rely on them, delivering an intervention aimed at disrupting a particular indicator that in turn puts the participant at risk of engaging in situations that could still trigger an episode (eg, suggesting someone to avoid texting a relative without knowing that this could make them anxious and in turn provoke an episode), and the long-term side effects of following certain interventions (eg, spending less time outside). Designing future solutions that actively consider responsible innovation and the possible negative consequences of certain app features will help avoid unintended consequences [1].

In the end, it is fair to assume that binge-eating monitoring and digital interventions will need to account for the frequency, life span, and computational amenability of the episodes’ indicators and find a trade-off between the severity and impact of such episodes and the goal, cost, risk, and the effectiveness of candidate interventions. This sensor-informed context could support people and their therapists in identifying the triggers of their binge-eating episodes or simply augment the nondigital strategies they already use. As with other forms of retrospective self-reporting [97-99], the consequences of showing participants historical contextual information, the validity of these reports, and cognitive biases that might come into play should be studied. To our knowledge, this is an open problem in binge-eating research.

### Usability and Acceptance

Although we wanted to know how to improve the functionality of the DeMMI app in our interviews, we did not set out to follow a usability engineering method for evaluating usability. Usability discussions were organically gathered from the interviews and identified as one of the themes in our qualitative analysis. At this early stage of our research, when we were simply collecting data as unobtrusively as possible, we wanted participants to have minimal tasks to complete when interacting with the app; they were only expected to log episodes and/or mood. Poor system usability has been highlighted as one of the factors affecting patient acceptance of health technologies [100]. As such, we envision the need to conduct usability evaluations such as heuristic evaluation and end user testing when the DeMMI app is further developed to include intervention-based functionality. End user testing examines how users conduct certain tasks or follow processes and is mainly focused on user experiences within the system [101]. Heuristics evaluation is conducted by usability experts and is concerned with the assessment of the system against a set of heuristic guidelines [102]. This type of usability engineering method would be an essential step when end users are expected to navigate through the app and engage with intervention-based content to support them in managing their disordered eating behaviors. Future work should adapt usability evaluation approaches taken, for example, by Honary et al [103] and Or and Tao [101], which are recommended for digital health solutions.

### Reflections on Approaches to Participant Engagement

Participant insights played a pivotal role in uncovering the link between mobile data and binge-eating occurrence. That said, participant engagement was a significant challenge that we experienced in this study. Our most successful recruitment medium was an Instagram campaign in which 177 people responded via email. However, only 15 (8.5%) people consented to participate after receiving the study information, and of those 15, only 10 (67%) installed our app. This low recruitment rate could be caused by concerns surrounding the sharing of mobile data that has the potential to expose web browsing and communication habits. However, as noted in both our consultation and poststudy interviews, and echoing the findings from the study by Honary et al [91], participants were generally satisfied with the information we had provided relating to how we would capture and use their sensor data through our *What You Do/What We See* resource, which we have made available for reuse (Multimedia Appendix 1). The clarity and transparency regarding what could be considered invasive data capture were enough to alleviate participants’ initial concerns, and we greatly suggest that future researchers use a similar approach in their research to increase participants’ literacy surrounding mobile sensed data. Furthermore, only 30% (3/10) of our binge-eating group participants agreed to be interviewed at the end of the study. This could be an exception, given our low number of participants but could also be linked to the shame and fear of stigma reported by those who binge eat [5]. Exit interviews are an important part of the research process; therefore, moving forward, we aim to explore the potential of questionnaire-based exit interviews that may be perceived by participants as more confidential.

### Limitations

This was an exploratory study aiming to explore the type of mobile sensing data that might be relevant for detecting episodes of binge eating. We acknowledge the limitations of our small sample size (20 participants), which makes it difficult to conclude any definitive findings, particularly given the individual differences between the participants. However, this preliminary study was conducted to inform the design of future larger-scale trials in this space. Given what we now know about participants’ engagement with the study, their acceptance of the methods, and their willingness to provide self-reported data, we are confident that a larger-scale study would be feasible. In addition, our study monitored a UK-only cohort of people with binge-eating behaviors, limiting our results’ generalizability to other contexts. During the study, our participants’ general behavior might not be representative of their usual routines because of mobility limitations during the COVID-19 lockdown. Our smartphone monitoring app was only compatible with Android and stopped collecting sensor data on 20% (4/20) of our participants’ phones, likely because of a software bug related to data synchronization, which, as it is, could limit the deployment of future studies using the same app. The exploratory nature of our study calls for the collection of data from multiple smartphone sensors. However, this might have influenced our low initial consent rate compared with the number of people initially interested in participating. Clearer study information materials provided early in the recruitment process and a more constrained sensing approach might alleviate this limitation.

### Conclusions

We conducted a preliminary analysis of the differences in smartphone-based behavioral features between days with and without binge-eating episodes to explore the feasibility of using mobile sensing to detect these events. We contextualized the experiences of people who binge eat and reflected on the challenges and opportunities for working with this population. In addition, we discussed the need to understand participants’ personal and social contexts preceding and accompanying their binge-eating episodes to be able to weigh the benefits, constraints, and risks of monitoring them using smartphones, as well as the implications of leveraging the insights extracted from these data sources to plan for safer and more effective digital interventions.

## Appendix

Multimedia Appendix 1 What You Do/What We See resource.

Multimedia Appendix 2 Installation instructions provided to participants.

## Abbreviations

CBT: cognitive behavioral therapy
DeMMI app: Detection of mental health behaviors using Mobile Interactions
EMA: Ecological Momentary Assessment
NAP: Nonoverlap of All Pairs
PANAS: Positive and Negative Affect Scale
RAPIDS: Reproducible Analysis Pipeline for Data Streams
