# Supplementary material for: Detecting Mental Health Behaviors Using Mobile Interactions: Exploratory Study Focusing on Binge Eating
Source: JMIR Ment Health. 2022 Apr 25;9(4):e32146. doi: 10.2196/32146 (PMC9086876; doi:10.2196/32146)
Supplement: Multimedia Appendix 1 [file mental_v9i4e32146_app1.pdf]

App notifications

What you do

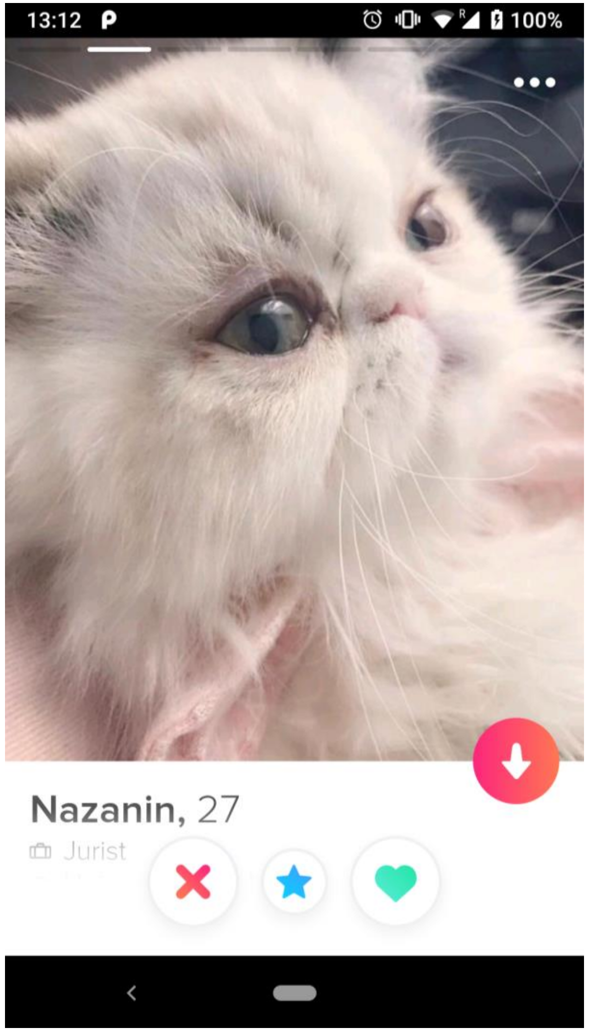

What we see

Participant

25124613-434b-4a02-906a-daee0cf8209d

Timestamp

19-March-2019 13:12:35

App

com.tinder.android

Notifications

19-March-2019 17:21:35

19-March-2019 17:32:35

19-March-2019 18:50:35

Crashes

20-March-2019 11:20:35

App use

What you do

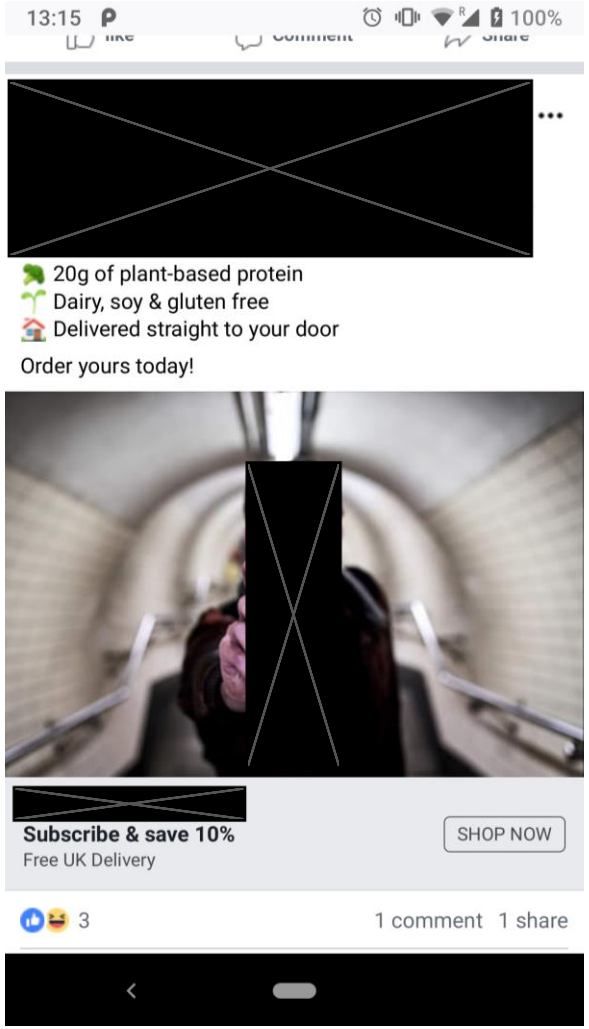

What we see

Participant

25124613-434b-4a02-906a-daee0cf8209d

Timestamp

19-March-2019 13:15:35

Event

Scroll, Tap

Source

Element 24

Target

Element 32

Typing

What you do

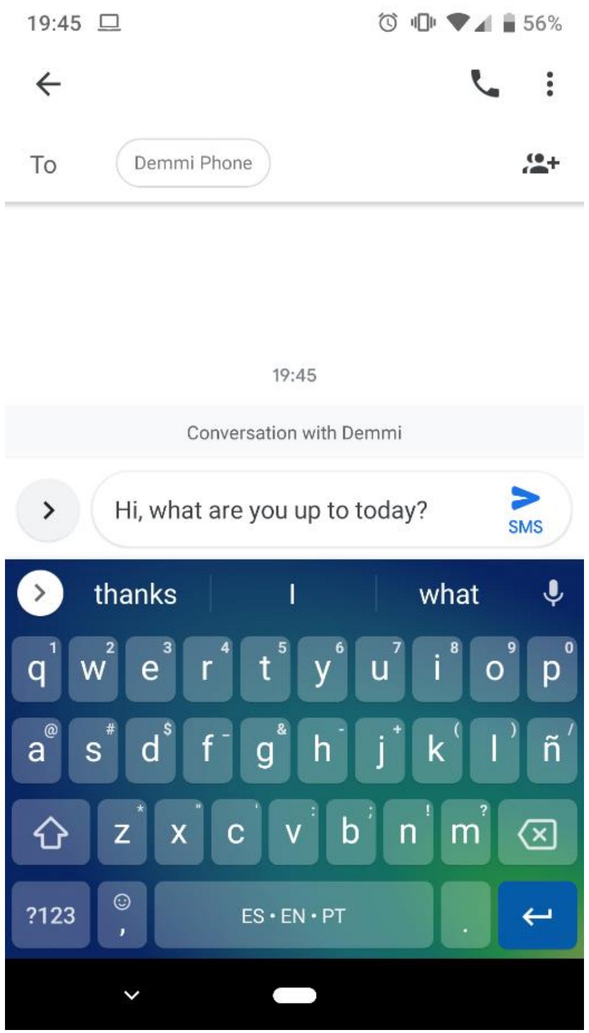

What we see

Participant

25124613-434b-4a02-906a-daee0cf8209d

Timestamp

19-March-2019 19:45:35

Key presses

Aa, aaaa aaa aaa aa aa aaaaa?

Battery

What you do

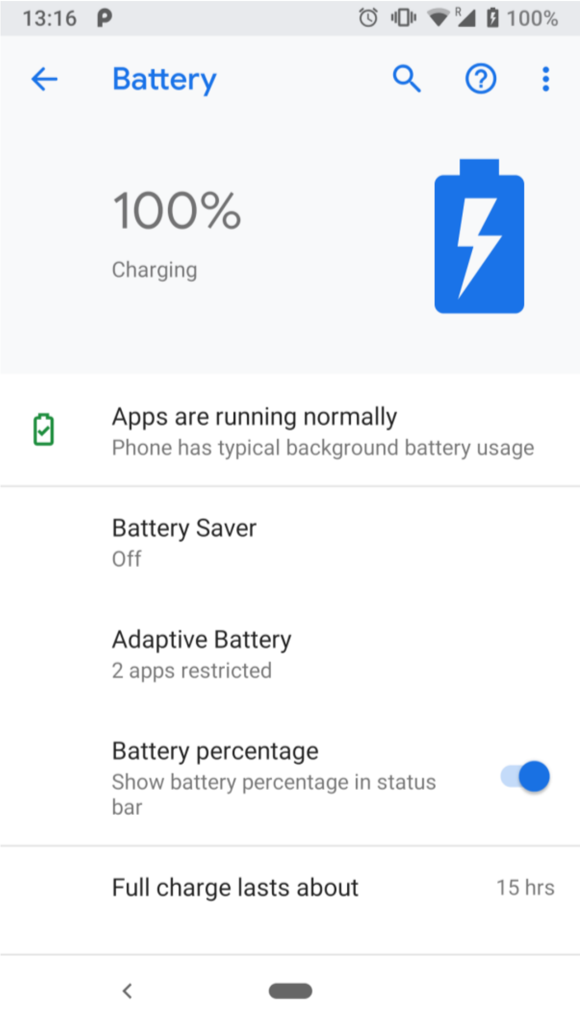

What we see

Participant

25124613-434b-4a02-906a-daee0cf8209d

Timestamp

19-March-2019 13:16:35

Battery level

0-100%

Calls

What you do

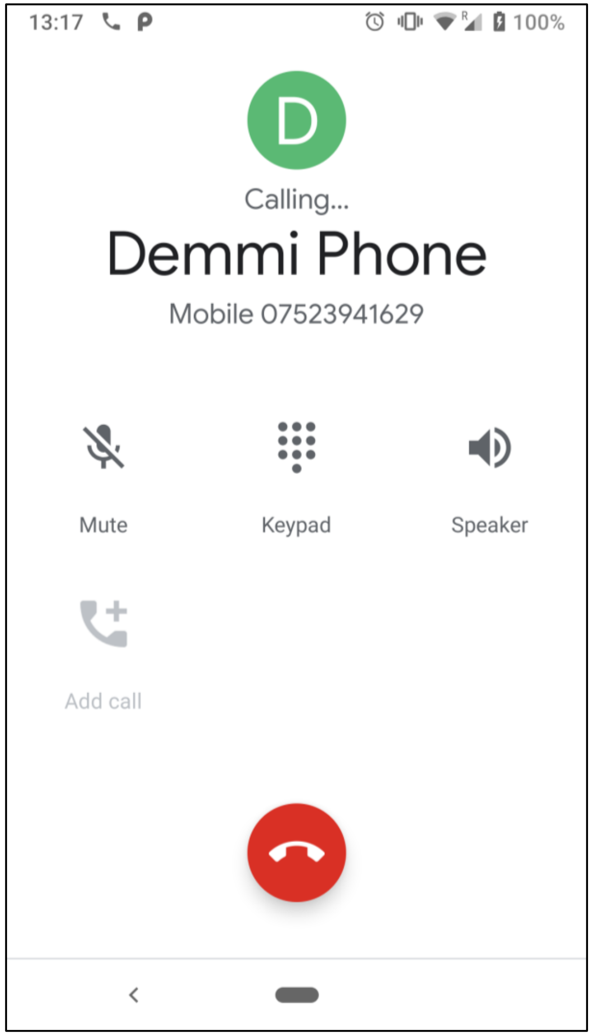

What we see

Participant

25124613-434b-4a02-906a-daee0cf8209d

Timestamp

19-March-2019 13:17:35

Person called

569b04f0ae1358560ac605fb1d11fd9d7c0d20

Duration

110 seconds

Type

Incoming/Outgoing

SMS

What you do

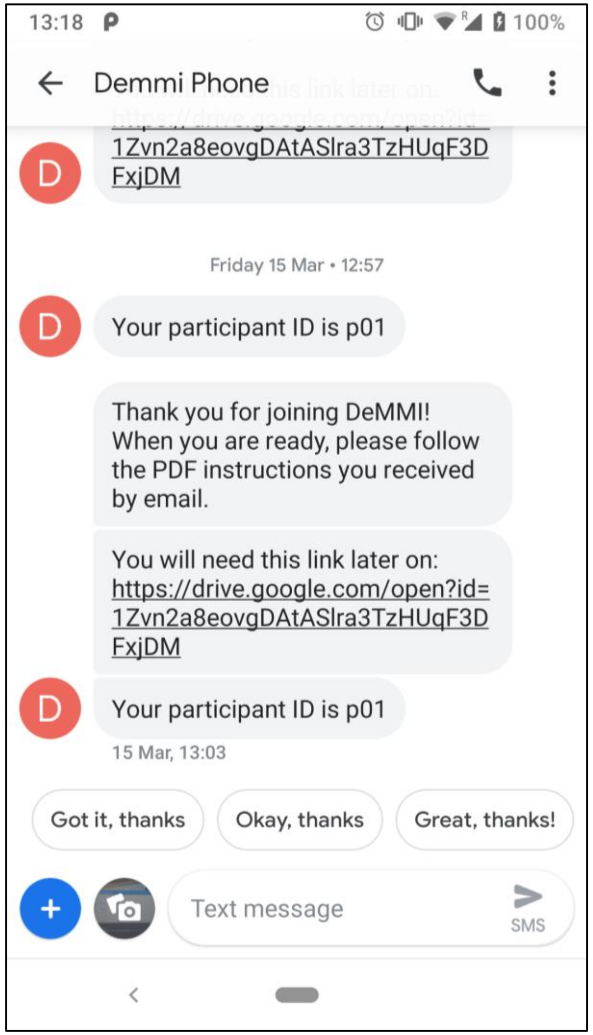

What we see

Participant

25124613-434b-4a02-906a-daee0cf8209d

Timestamp

19-March-2019 13:18:35

Person texted

569b04f0ae1358560ac605fb1d11fd9d7c0d20

Type

Incoming/Outgoing

Data sent/received

What you do

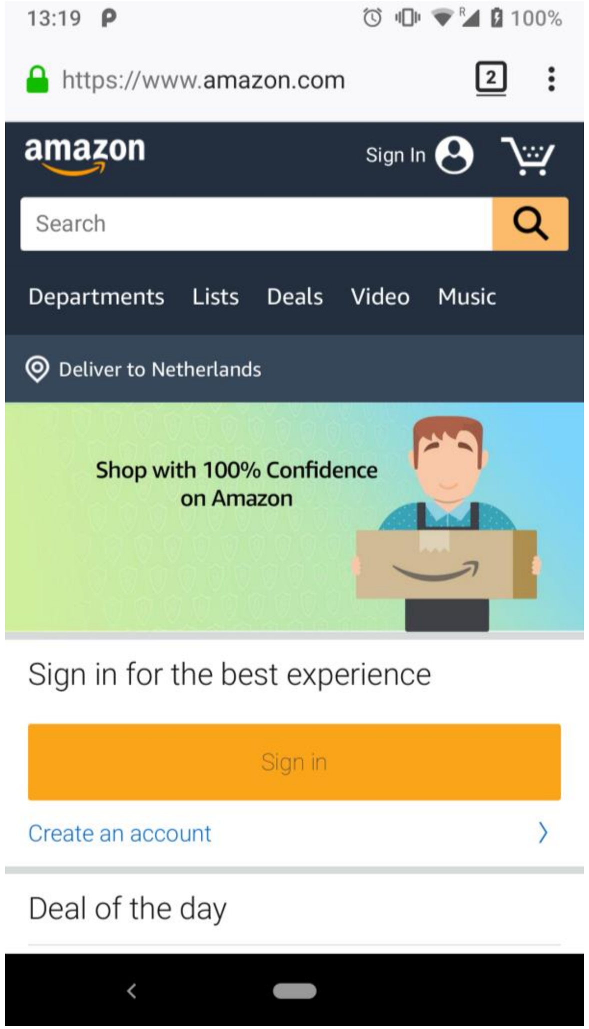

What we see

Participant

25124613-434b-4a02-906a-daee0cf8209d

Timestamp

19-March-2019 13:19:35

Sent/received bytes (10101101)

a number (0-99999)

Sent/received packets

a number (0-99999)

Screen locks/unlocks

What you do

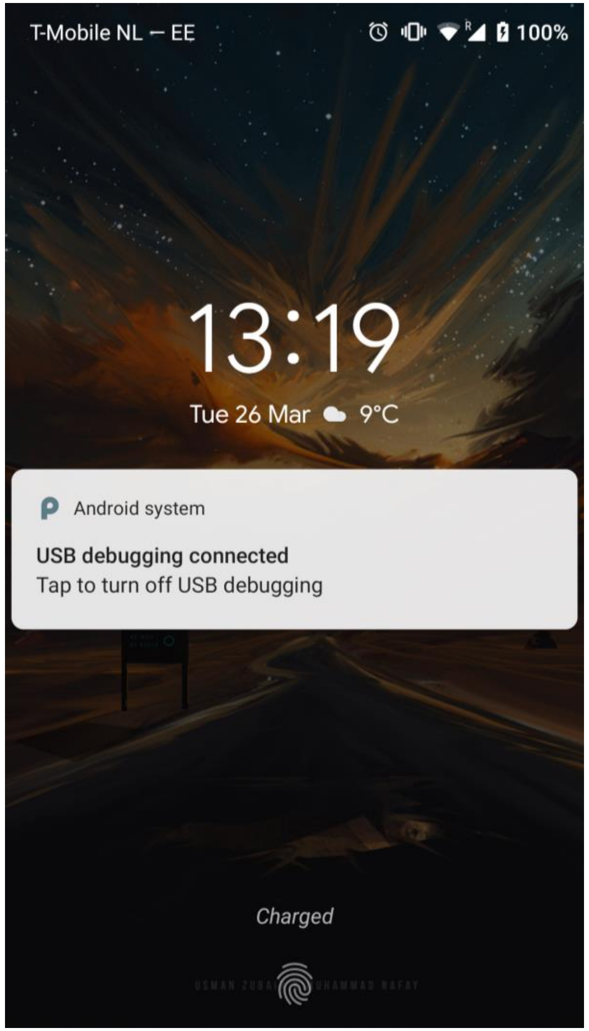

What we see

Participant

25124613-434b-4a02-906a-daee0cf8209d

Timestamp

26-March-2019 13:19:35

Screen event

ON, OFF, Unlock, or Lock

Time zone

What you do

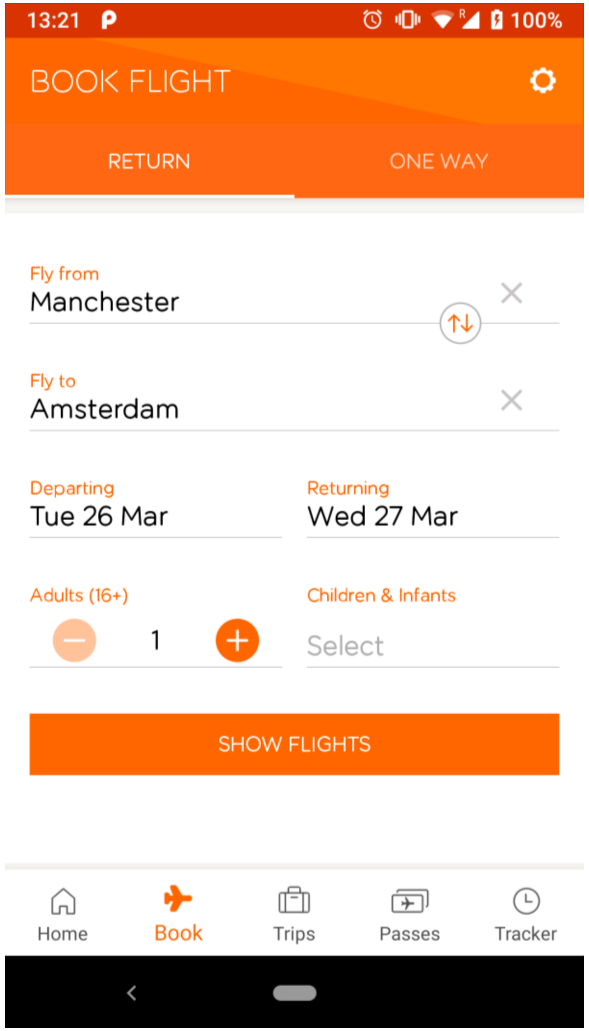

What we see

Participant

25124613-434b-4a02-906a-daee0cf8209d

Timestamp

26-March-2019 13:21:35

Time zone

Central European Summer Time

Wi-Fi

What you do

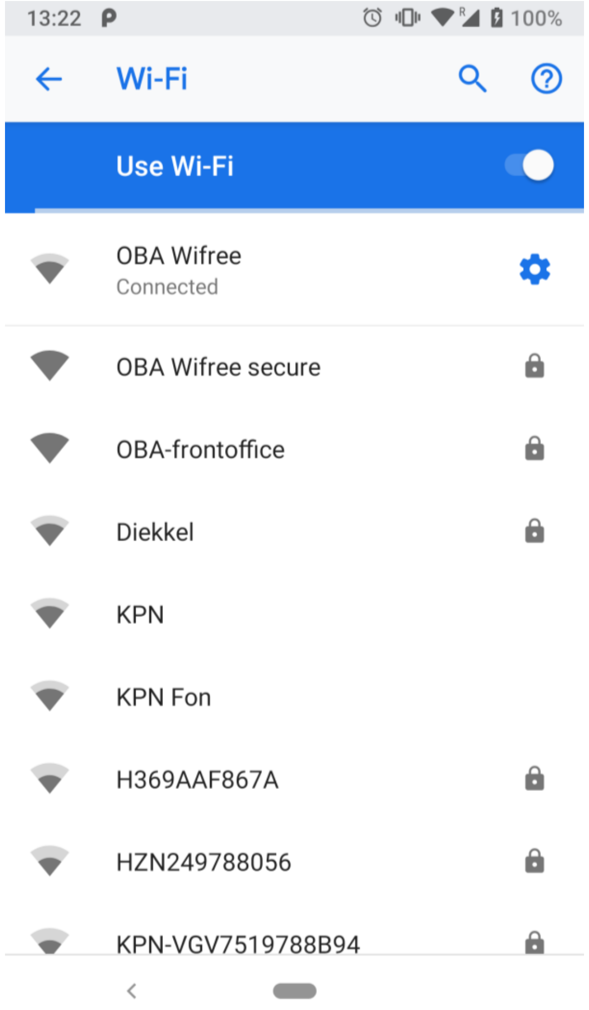

What we see

Participant

25124613-434b-4a02-906a-daee0cf8209d

Timestamp

19-March-2019 13:22:35

Wi-Fi access point label

Example: eduroam, The Cloud, or Costa

Bluetooth

What you do

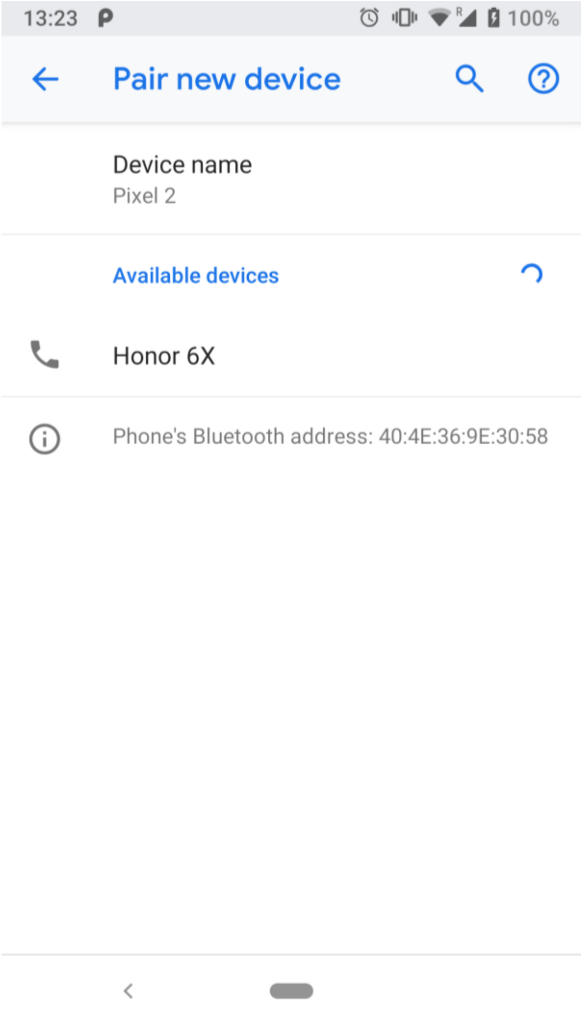

What we see

Participant

25124613-434b-4a02-906a-daee0cf8209d

Timestamp

19-March-2019 13:23:35

Device label

Example: Honor 6X, or Richard's, or Fitbit

Light

What you do

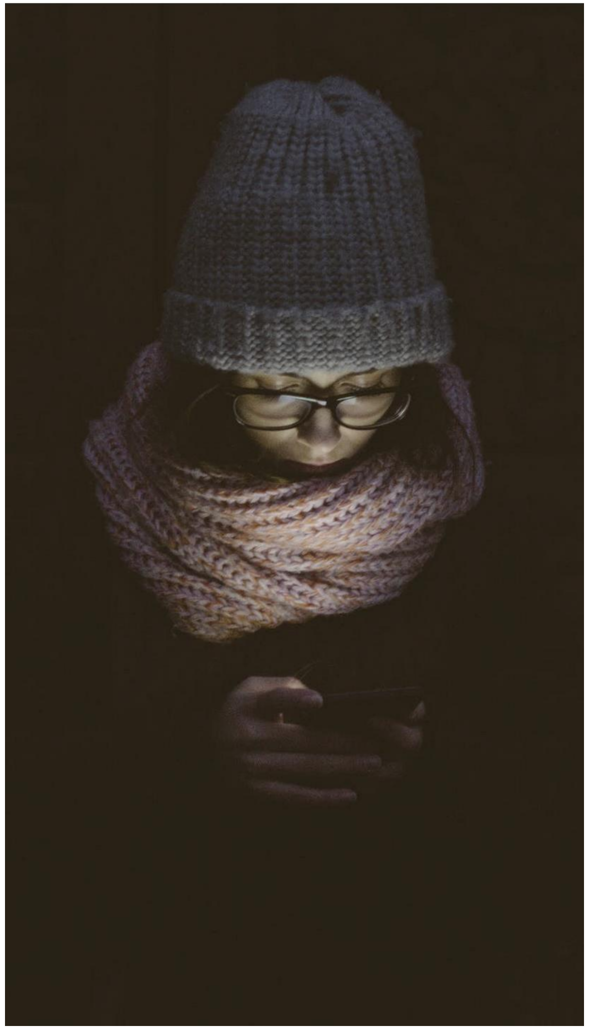

What we see

Participant

25124613-434b-4a02-906a-daee0cf8209d

Timestamp

19-March-2019 19:00:35

Light level

0-255

Weather

What you do

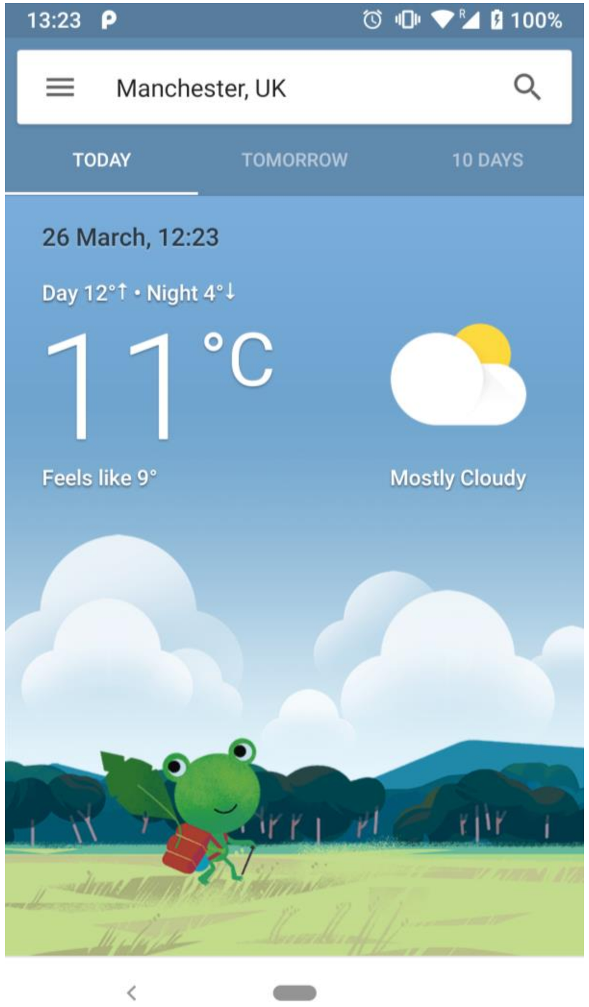

What we see

Participant

25124613-434b-4a02-906a-daee0cf8209d

City

Bristol

Temperature

15 C

Pressure

122

Humidity

75%

Wind Speed

15 km/h

Conditions

Cloudy/Rain/Snow

Sunrise/Sunset

07:19/18:14

Description

Heavy Rain

Location

What you do

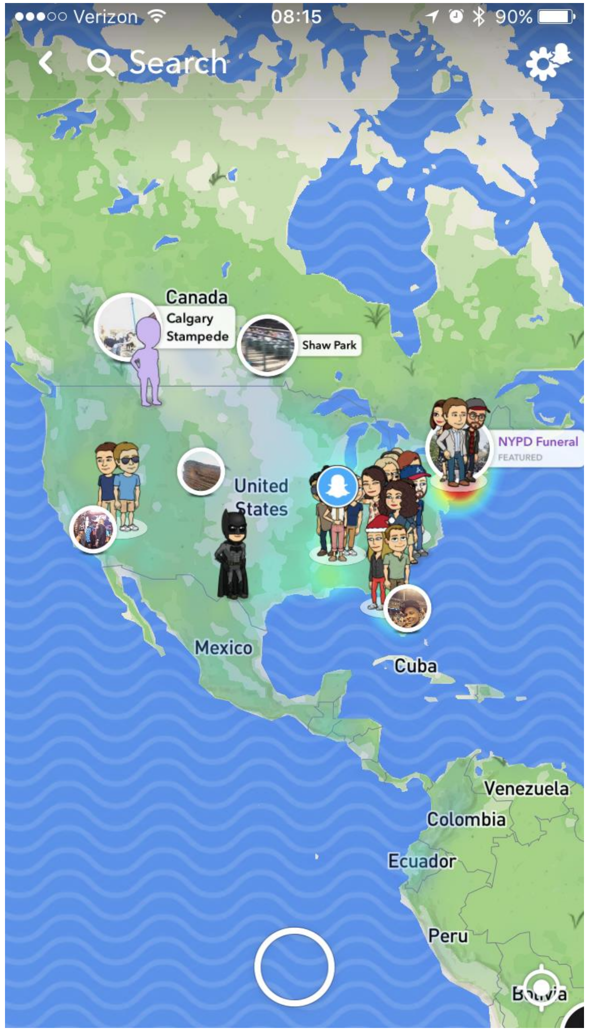

What we see

Participant

25124613-434b-4a02-906a-daee0cf8209d

Timestamp

19-March-2019 08:15:35

Latitude

51.45667

Longitude

-2.25997

Ambient Noise

What you do

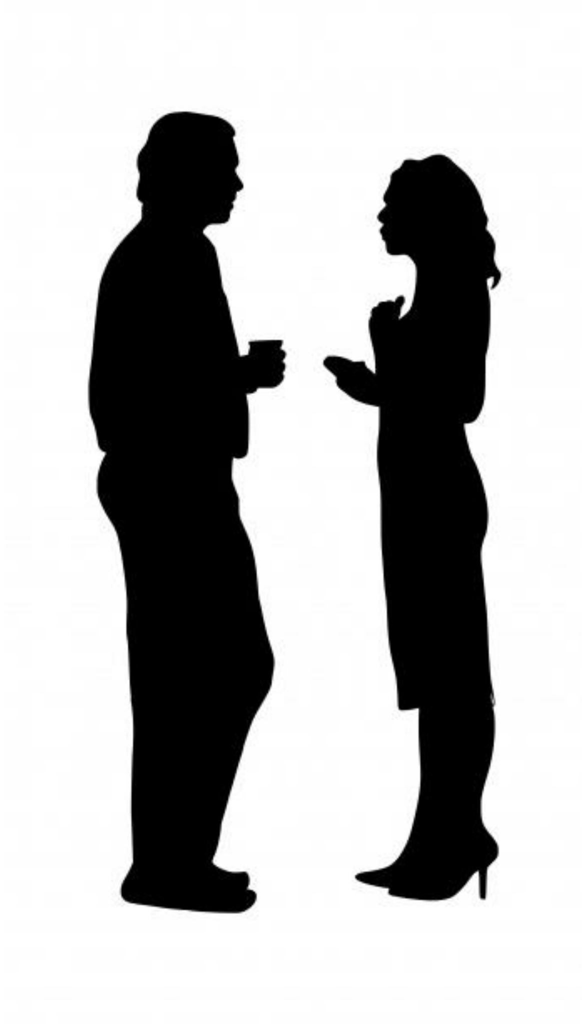

What we see

Participant

25124613-434b-4a02-906a-daee0cf8209d

Timestamp

19-March-2019 19:00:35

Decibels

65

Silent?

Yes/No

Activity Recognition

What you do

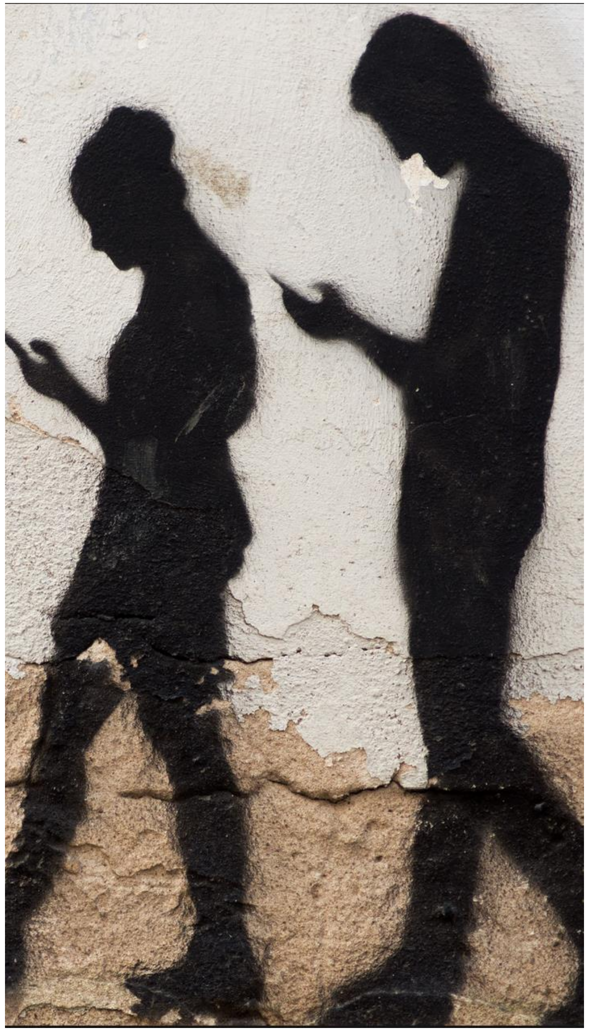

What we see

Participant

25124613-434b-4a02-906a-daee0cf8209d

Timestamp

19-March-2019 16:00:35

Activity

Running/Walking/Still/In-Veihcule/Cycling
