# Supplementary material for: Detecting Mental Health Behaviors Using Mobile Interactions: Exploratory Study Focusing on Binge Eating
Source: JMIR Ment Health. 2022 Apr 25;9(4):e32146. doi: 10.2196/32146 (PMC9086876; doi:10.2196/32146)
Supplement: Multimedia Appendix 2 [file mental_v9i4e32146_app2.pdf]

DEMMI

PHONE DATA COLLECTION REMINDER

We will collect the following data from your phone using the AWARE app:

- Phone movement (accelerometer)
- Screen and battery status
- Call and text events
- Approximate location of the phone
- List of currently running applications
- Nearby Bluetooth and WiFi connections
- Keyboard typing events
- Physical activity
- Ambient light

We are **only collecting meta-data** (basic descriptions of the data being collected e.g. Facebook is being used for 30 minutes, rather than what you are actually looking at on Facebook). **No identifiable information** will be collected through this app.

We will **not see or record** your contact names or numbers, your internet browsing history, the content of the apps you use, nor the text you type (just timestamps)

INSTALLING AWARE (1)

1. On your phone's internet browser, go to **bit.ly/appdemmi** to download the AWARE app
2. If necessary, tap **"Download"** on the prompt at the bottom of screen
3. Tap **"Open"** when download is complete
4. If there is a popup that says your phone is not allowed to install unknown apps from this source, follow the link to go to your phone's settings to Allow downloads from this source.

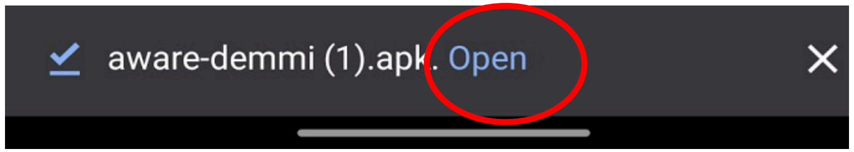

INSTALLING AWARE (2)

1. Tap "**Install**" on the next screen
2. After AWARE has been installed, tap "**Open**"
3. When prompted, tap "**Allow" for all permissions prompts** (contacts; phone calls; pictures and video; location; photos, media, and files)

   NOTE. These permissions are required by AWARE. However, we only collect the data mentioned before. We **will not** collect info on contacts, pictures, videos, photos, media, or files on your phone.
4. If asked for when AWARE can access **location**, tap "**Allow all the time**"
5. If asked to "**Stop optimizing battery usage**", tap "**Allow**"
6. If asked, tap "**Allow**" for **background permission**

JOIN THE STUDY

1. On your phone's internet browser go to the link we emailed you: **https://demmi.info/join.html…**
2. Tap on **"Join the study!"**

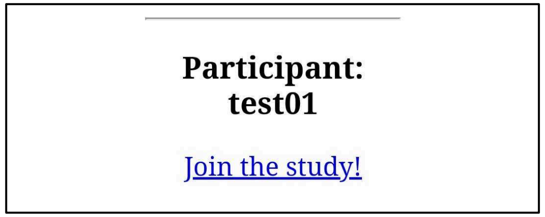

3. Then tap on the **"Join study"** prompt to open the AWARE app

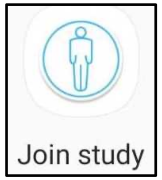

4. Tap **"Sign up!"**
5. **Accept all permissions** that come up (e.g., Bluetooth, call logs, SMS messages)
6. **Swipe down to see notifications**
7. Tap the notification that says **"Please enable AWARE"**
8. This will redirect you to Accessibility settings, where you should tap **AWARE** (may be under "Installed services") and turn the setting to **"On"**

SETUP YOU SYNCING PREFERENCES

1. Open the **AWARE app**
2. Tap **Sensors** at bottom left

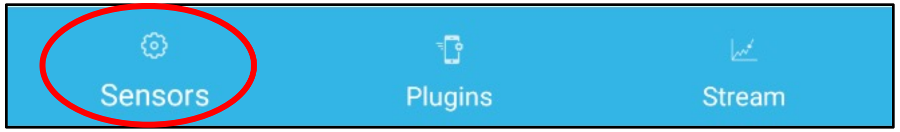

3. Scroll down and **tap “AWARE Study” (study and sync settings)** near the bottom of the page
4. For **“Wifi only”** choose **On** or **Off:**
   - **On** (checked box) if you can connect to a WiFi network at least once per day
   - **Off** (unchecked box) if you cannot AND are **able** to use an additional 1GB of data per week
5. **Leave all other settings as they are**

REPORTING YOUR MOOD

We ask you to report your mood every day at 9:00 am and 9:00 pm:

1. The screen on the right will be shown automatically at those times
2. **Tap on the emoji** that best describes your mood at that moment

NOTE: To report your mood more than twice a day, swipe down to see your notifications, swipe down again on the DeMMI notification and tap **"LOG MOOD"**

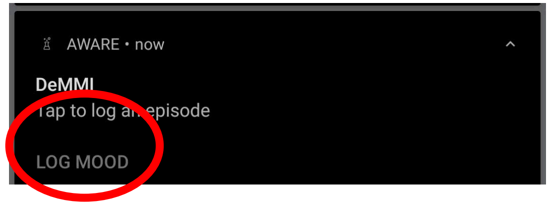

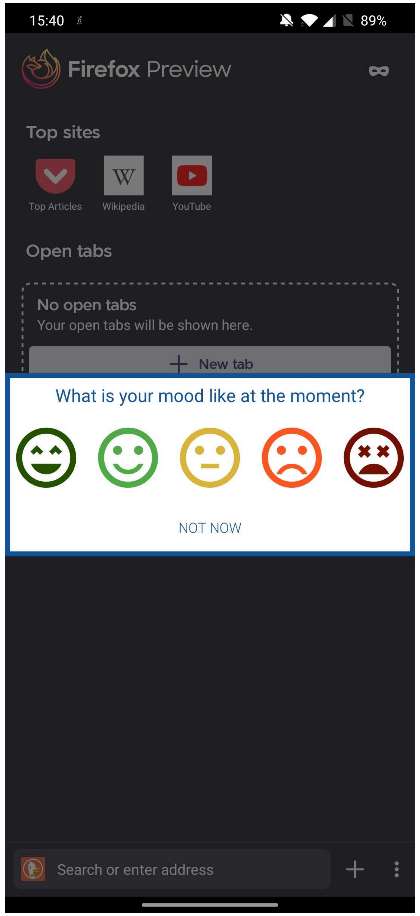

REPORTING AN EPISODE

After you go through a an episode that you would consider to be harmful (e.g. self harm, binge eating episode, episode of purging), please let us know by:

1. Swiping down to see your notifications and tapping **"Tap to log an episode"**

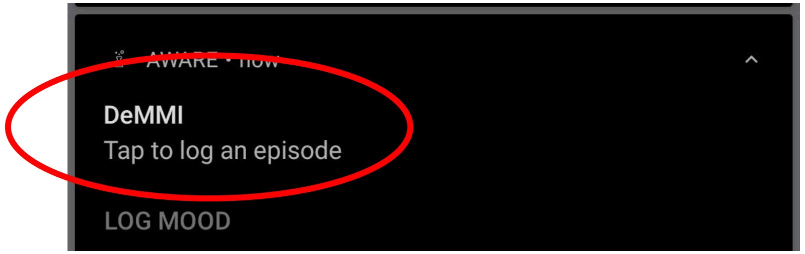

2. Add any optional comments and tap the **"LOG EPISODE"** button

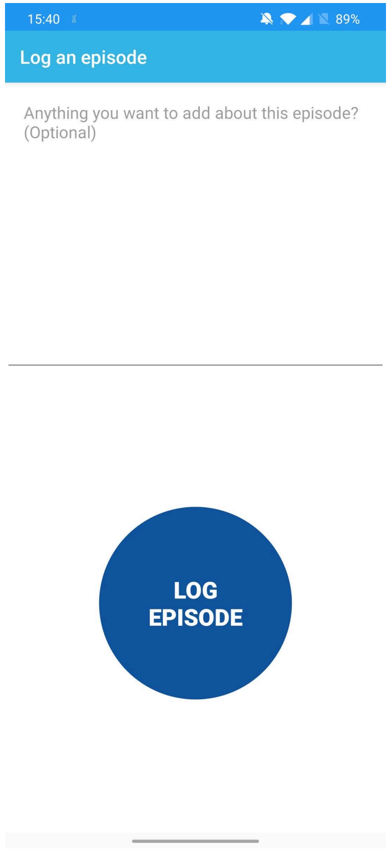

THINGS TO REMEMBER

- During the study, **you do not need to go into the AWARE app.**
- **Don't stop the AWARE** app by swiping up on it. You will know it is running if you can see the notification to report your mood and episodes.
- If possible, **try to avoid putting your phone in battery saver mode** as data will not be collected during this time
- **If the AWARE app crashes** or you see a message saying the app stopped working, close that message. If the app crashes constantly try restarting your phone

DELETING THE AWARE APP

You can uninstall the Aware app just like any other app in your phone

When you remove the app, all collected data will be deleted from your phone
